# Broad consent in the emergency department: a cross sectional study

**DOI:** 10.1186/s13690-025-01529-z

**Published:** 2025-02-18

**Authors:** Antje Fischer-Rosinský, Larissa Eienbröker, Martin Möckel, Frank Hanses, Felix Patricius Hans, Sebastian Wolfrum, Johannes Drepper, Philipp Heinrich, Anna Slagman

**Affiliations:** 1 Health Services Research in Emergency and Acute Medicine, Charité - Universitätsmedizin Berlin, Berlin, Germany; 2 Emergency Department, Department for Infection Control and Infectious Diseases, University Hospital Regensburg, University Hospital Regensburg, Regensburg, Germany; 3 Medical Center, Faculty of Medicine, University Emergency Center, University of Freiburg, University of Freiburg, Freiburg, Germany; 4 Emergency Department, University Hospital of Schleswig-Holstein, Campus Luebeck, Luebeck, Germany; 5 TMF–Technology, Infrastructure for Networked Medical Research, Methods, Berlin, Germany; 6 Faculty of Medicine Carl Gustav Carus, Unabhängige Treuhandstelle, Technische Universität Dresden, Dresden, Germany

**Keywords:** Broad consent, Feasibility, Informed consent, Emergency department, Patient-reported voluntariness, Recall, Motivation, Satisfaction

## Abstract

**Background:**

The Medical Informatics Initiative (MII) introduced a broad consent form (MII-BC) encompassing clinical, insurance, and biomaterial data, along with re-contacting options. In the emergency department (ED), outpatient and inpatient patients of all illnesses and severity could be reached early in their treatment course. The BC-ED (Broad Consent in the Emergency Department) project uniquely investigated the implementation of MII-BC in EDs, exploring feasibility, selection bias and patients’ perceptions of voluntariness, information recall, motivation, and satisfaction.

**Methods:**

The BC-ED project involving four university hospital EDs in Germany, is part of CODEX+ (Collaborative Data Exchange and Usage), an initiative within the Network University Medicine (NUM). To minimize selection bias, a systematic sampling approach (every 5th/30th patient) was applied, with patient recruitment and consent processes adapted to local conditions and therefore varying among sites. Data collection included patient questionnaires, surveys completed by study nurses, and routine clinical data. Analysis was conducted descriptively using SPSS.

**Results:**

Of 1,138 patients approached, 553 (48.6%) were capable of giving consent. Of 353 patients who could not consent, primary reasons included language barriers (35.4%) and inability to grasp study details (21.5%). Of all eligible patients, 3.3% could not be contacted. Of 535 (47.0%) patients able to consent and contacted, 313 consented to the MII-BC. Resulting in a consent rate of 27.5% corresponding to the baseline population and 58.5% of those contacted. Motivations for consenting were general support for research (85.3%) and the desire to help future patients (78.2%). Patients generally reported a high level of understanding and satisfaction with the consent process, reporting comprehensive understanding of scientific data use (89.8%) and associated risks (82.2%). However, discrepancies were noted between consented options and patient recall.

**Conclusions:**

This study is the first to investigate the implementation of the MII-BC in the challenging ED environment. With a consent rate of 27.5% total baseline population and 58.5% of those contacted, it demonstrates that patients were able and willing to participate in research. Reasons for non-consent were barriers like language and medical conditions. Strategies to address these barriers are crucial for inclusivity. Although patients generally understood the consent process, discrepancies in recall highlight the need for improved comprehension strategies.

**Trial registration:**

German Clinical Trials Register on 25 October 2022 (DRKS0003054).

**Supplementary Information:**

The online version contains supplementary material available at 10.1186/s13690-025-01529-z.

**Table:** 

Text box 1. Contributions to the literature	
• This is the first study addressing the consent rate and feasibility of the Broad Consent (BC) in the emergency department (ED) setting. • We found a relevant consent rate of 27.5% of all patients treated and 58.5% of those who were approached regarding BC. • The advantage of using BC in the ED is a reduced selection bias due to access to patients of all disease and socio-demographic entities, as well as the possibility to obtain early patient consent before it may no longer be possible due to changes in health status.	

## Introduction

For both research and patient care, it is of great importance to create comprehensive infrastructures for data collection. Health data play a critical role in advancing medicine and improving prevention and treatment. Optimising the use of health data is therefore essential to improve patient outcomes and the overall efficiency of the healthcare system [1]. Countries such as the UK, with its National Health System (NHS), have established interoperable networks for healthcare data. The NHS is made up of many inter-connected organisations that work together to enable complex interactions and data sharing between healthcare providers, systems and patients. In the UK, most patients registered with NHS services are part of ongoing projects where their health data is shared between different healthcare providers in their area [2].

The informed consent of patients, among other things, is a possible legal basis for the broad transmission and linking of medical data in centralised structures (Article 9 part 2a EU General Data Protection Regulation, GDPR). Alternatively, a national legislator (or the EU) may create a legal basis for the processing of health data for research purposes on the basis of Art. 9 part 2j GDPR. Self-determination in data donation for secondary use can be achieved through opt-in or opt-out procedures. Opt-in requires explicit consent before data processing begins, while opt-out requires explicit refusal. Opt-out solutions are ultimately not consent-based either, but a legal basis for use has been created that merely provides for an opt-out as a supplementary organizational measure, for example [3]. In Europe, opt-out procedure is used by a number of countries, including Austria, France, Poland and Spain [4]. At the time of the study, there was no transnational law in Germany that would have allowed the centralised consolidation of health data from routine care, so informed consent in the form of an opt-in was the only option for this measure. An example of this is the Broad Consent (BC) project, currently being carried out in Germany as part of the German Federal Ministry of Education and Research (BMBF)-funded Medical Informatics Initiative (MII) [5]. Core characteristic of this BC is the EU-GDPR-compliant combination of the principle of informed consent with the absence of a specific research purpose (compared to study-specific consents).

The MII aims to provide digital, reliable and rapid access to routine health care data for medical research. The MII pursues a decentralised storage approach in which patient care data is stored where it is generated. This data is then used for various medical research purposes via networked data integration centres. These centres are operated by the German university hospitals and other collaborators, organised in consortia [6, 7]. The MII has designed a BC form that comprehensively includes different consent modules: clinical treatment and insurance data of patients as well as biomaterials and the possibility of re-contacting study participants for further study purposes [8]. 

The BC is already used at most university sites in Germany for patients who are admitted as inpatients, rarely in an outpatient setting. This approach involves an unavoidable selection bias introduced by the inpatient admission as the primary inclusion criterion. In the ED more than 50% of the patients are outpatients who are easily accessible in this setting.

To recruit patients early while accessing the health care system through the emergency department (ED) would facilitate reaching a broader spectrum of patients (e.g. outpatients, hard to reach vulnerable patients, patients with all levels of disease severity, patients who might deteriorate and may be incapable of giving consent at a later stage) and thus reduce selection bias. Ethical considerations have to be considered regarding the ability to give informed consent in the ED setting, such as a severe degree of illness, sedated patients and severe pain. To date, only very limited evidence exists on the general feasibility of obtaining BC in the ED. The implementation of BC in the ED setting would provide access to a broad, heterogeneous patient population and offer immense benefits for health data research.

Thus BC-ED was conducted to evaluate the feasibility, selection bias and resources needed to obtain BC in the ED. The project aimed to evaluate patient-related and organizational factors influencing the obtainability of the BC in the ED, as well as voluntariness, information recall, motivation and patient satisfaction related to the BC process.

## Methods

### Study setting

BC-ED is a sub-project of CODEX+ (Collaborative Data Exchange and Usage) which was intended to expand the collaborative infrastructure of CODEX from the Network of University Medicine (NUM). The aim of CODEX + was to develop organisational and technical solutions to enable all partners to collect, exchange and analyse data together in terms of the network’s pandemic preparedness [9]. It was funded by the Federal Ministry of Education and Research (01KX2121). The EDs of the four university hospitals located in Berlin, Freiburg, Regensburg and Lübeck in Germany participated in the project during the recruitment period.

### Patient recruitment and study design

**Fig. 1:**
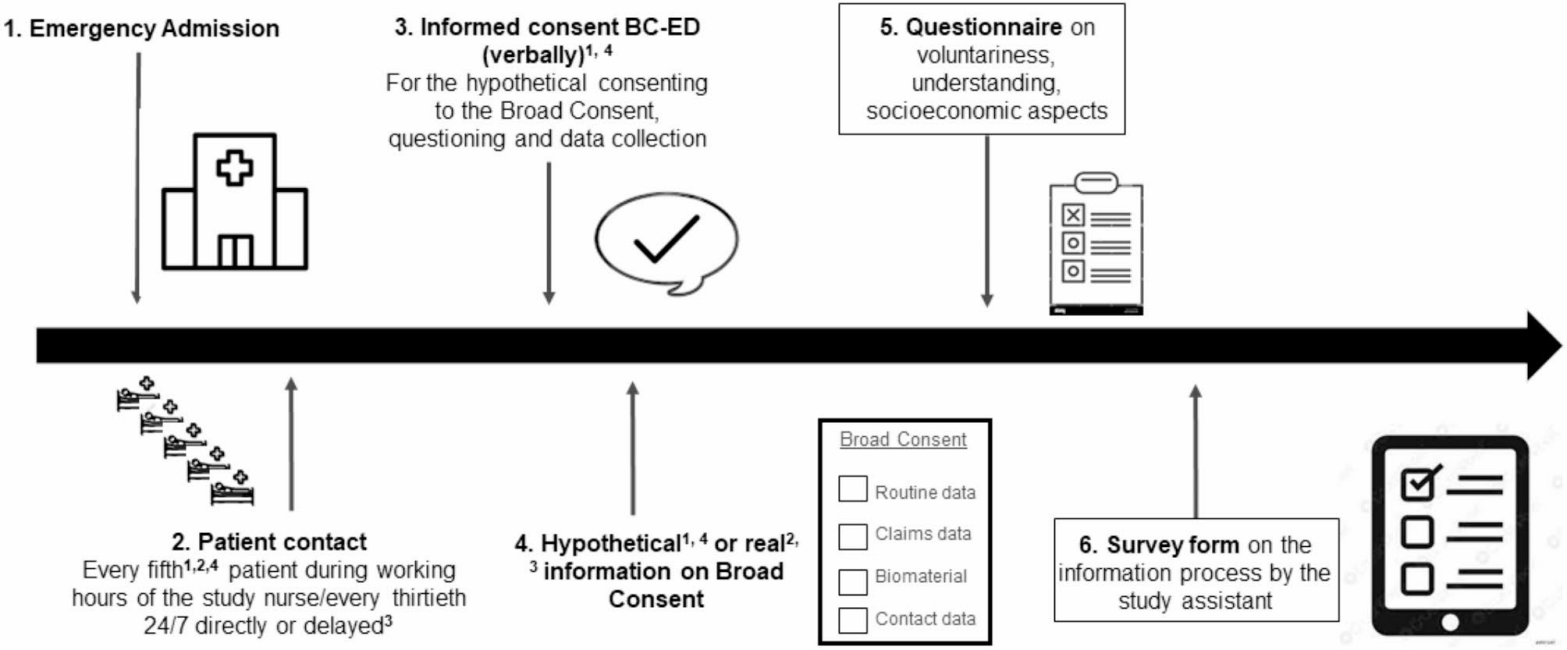
Study design of the BD-ED project. BC-ED– broad consent in the emergency department. Study sites were: 1– Charité - Universitätsmedizin Berlin, 2– Universitätsklinikum Regensburg, 3– Universitätsklinikum Freiburg, 4– Universitätsklinikum Lübeck

Recruitment at all ED sites took place from September 2022 to December 2022. Patients were eligible for inclusion if they were 18 years of age or older and capable of giving consent in respect of their medical and cognitive condition. In order to prevent selection bias and to reflect the accessibility of patients of all severity levels in the ED, a systematic sampling approach was employed, where every fifth patient was pragmatically approached in Berlin, Regensburg and Lübeck according to the administrative admission time within working hours of the study nurse. In Freiburg every thirtieth patient admitted to the ED was approached 24/7. At this location the patients were contacted “delayed” either by telephone or on the ward, whereas all other locations contacted patients directly in the ED [10]. In Regensburg and Freiburg, the MII-BC could actually be implemented “for real” as the regulatory (e.g. ethics) and structural requirements (e.g. data trust center, data warehouse) were given. In Berlin and Lübeck the BC was obtained “hypothetically” due to lack of regular and structural requirements. At those study sites the first step was to obtain verbal approval for participating in the BC-ED project itself and, in a second step, the hypothetical consent to the BC was obtained. Information on BC was provided via the detailed consent form version 1.7.2 (Supplement 1, only available in German language) and/or by providing the information video from the MII [11]. After the information about the content of BC, the modular consent form was filled out. In Regensburg the module adressing biomaterial was excluded as this was not covered by the ethics approval at this study site. Afterwards the study nurse entered the information regarding the consented modules of the MII-BC in the electronic documentation system (electronic case report form– eCRF, system used: REDCap^®^). Additionally, patients were asked to complete a questionnaire and the study nurse completed a survey form pertaining to the consent process, which was mentioned by the study nurse at the same time when introducing the consent procedure. The representativeness of a sample in studies in the challenging ED setting is expectedly limited and thus generalisability. The corresponding reasons are recorded via the survey of the study nurses. The study design is shown in Fig. 1.

### Study procedures and data collection

Patients consented in the different modules of the MII-BC, which was transferred by the study nurse into the eCRF in REDCap^®^. Further the patients have been asked to answer a questionnaire which addressed understanding, comments on the MII-BC itself or the consenting process, motivation, recall, voluntariness, participation in other studies, employment in the healthcare system, sufficient information and reflection time. Furthermore, sociodemographic information was obtained: age, gender, country of birth, spoken language, education, employment, life circumstances (Supplement 2, English and German version [12–14]). The questionnaires were completed paper-based and later transferred to the eCRF or entered directly electronically via tablet. The questionnaire and survey were developed by the study team and piloting was carried out on approximately four patients at the centers. The questionnaires, survey and further processes were adapted based on the results.The study nurse completed a survey form addressing the general procedure and features of the screening and consent process containing information on reasons for inability, unavailability and unwillingness to consent. In addition, time of beginning and end of the consent process, interruptions or terminations and respective reasons, which modules were consented to, reflection time requested, location, information material, and any additional comments were documented (Supplement 3, English and German version). To ensure consistency in data documentation, weekly meetings with the study nurses from all ED sites were conducted to clarify questions about inclusion criteria and discuss difficult cases.

The development of both the questionnaire and the survey form is mainly based on a review by Gillies et al. [15] and other literature [12, 13, 16] as well as personal exchange with Philipp Heinrich from the TU Dresden and standard instruments of the study site at Charité– Universitätsmedizin Berlin. The questionnaires were discussed and finalized in consultation with all four study sites. Due to the sensitivity of some questions and in order to be able to delimit whether the answer was intentionally omitted or not, the option “prefer not to answer” was inserted.

The modules for which the patient consented in the MII-BC have been transferred from the paper-based version by the study nurse or directly entered by the patient via tablet. Also, after completing the consent process, the patient has been asked via the questionnaire for which modules she or he has been consented to. This approach made it possible to check whether the information in the signed consent form matched the answers in the questionnaire completed after consent was given.

### Sample size calculation

As the study is investigating the feasibility of BC in the ED context, no specific sample size planning was carried out. Within the tight timeframe of the project, a sample size of approximately 100 patients per site was estimated, for a total of 400. With this target case number, precise estimates are possible at a prevalence of 50% with a precision of ± 8.04.9% (half the width of a two-sided exact 95% confidence interval). A prevalence of 50% was chosen because it shows the greatest variation. Higher or lower prevalences would lead to a more precise estimate.

### Statistical analysis

After data assessment in REDCap^®^, data were exported, quality checked and analysed using SPSS version 27. To ensure survey anonymity, all personally identifiable information was removed from the dataset prior to analysis. There were some additional free text options (for example, to indicate the reasons for non-participation and non-contacting), which were categorized accordingly for the evaluation in the cross-check by two evaluators. Absolute frequencies and relative proportions are shown for categorical data, continuous variables are presented as median and inter quartile ranges (IQR). Analysis have been descriptive and of explorative nature. Missing data have been considered and reported in the results to ensure transparency and completeness of data presentation. There was no replacement of missing values e.g. due to imputation. In the study, quantitative research standards were followed to ensure the quality and transparency of the research. The Checklist for Reporting Of Survey Studies (CROSS) [17] is added as Supplement 4.

### Ethical considerations

The study was approved by the institutional review boards of the four university sites (EA1_135_22–Charité– Universitätsmedizin Berlin; 22-1202-S1 - Freiburg; 22-3024-101–Regensburg; 2022 − 500–Lübeck). Data protection was approved by the clinical trial office (CTO) and data protection officers at Charité– Universitätsmedizin Berlin.

## Results

### Study population - screening, contacting and consenting patients

**Fig. 2:**
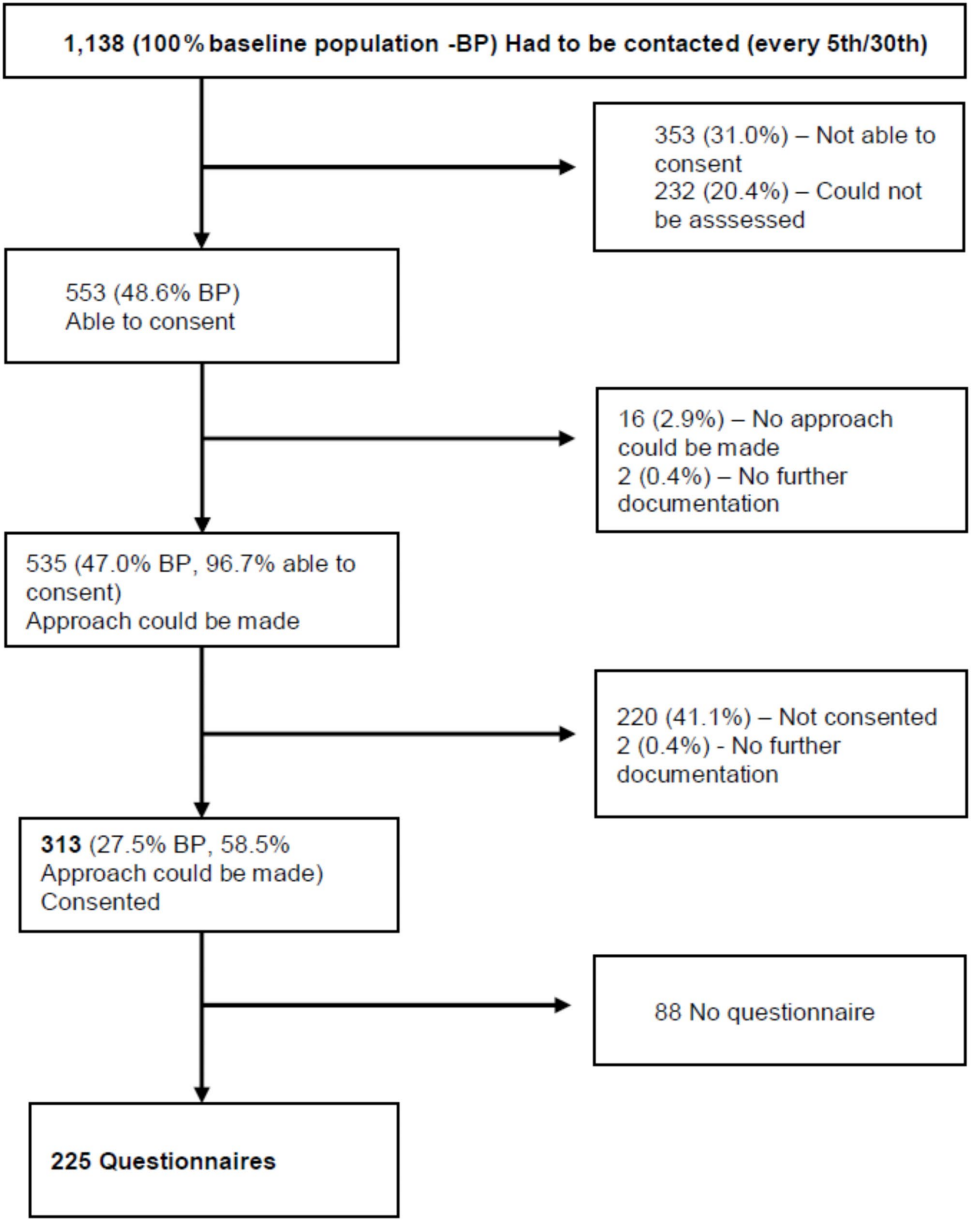
Flow chart of the patients to be contacted according to their ability to consent, responsiveness and final consent. BP– Baseline population, reasons for “Not able to consent” are shown in Fig. 3, reasons for “No approach could be made” are shown in Fig. 4, reasons for “Not consented” are shown in Fig. 5

A total of 1,138 patients had to be approached for BC in the ED. Of these, 553 patients were capable of giving consent (48.6%). Patients whose ability to consent could not be assessed were not seen by the study nurse (20.4%), but were included in the list in accordance with the pragmatic approach of contacting every fifth or thirtieth patient. 535 patients were approached by the study nurses (47.0%). Finally, 313 patients consented (27.5% of the baseline population and 58.5% of approached patients) (Fig. 2).

**Fig. 3:**
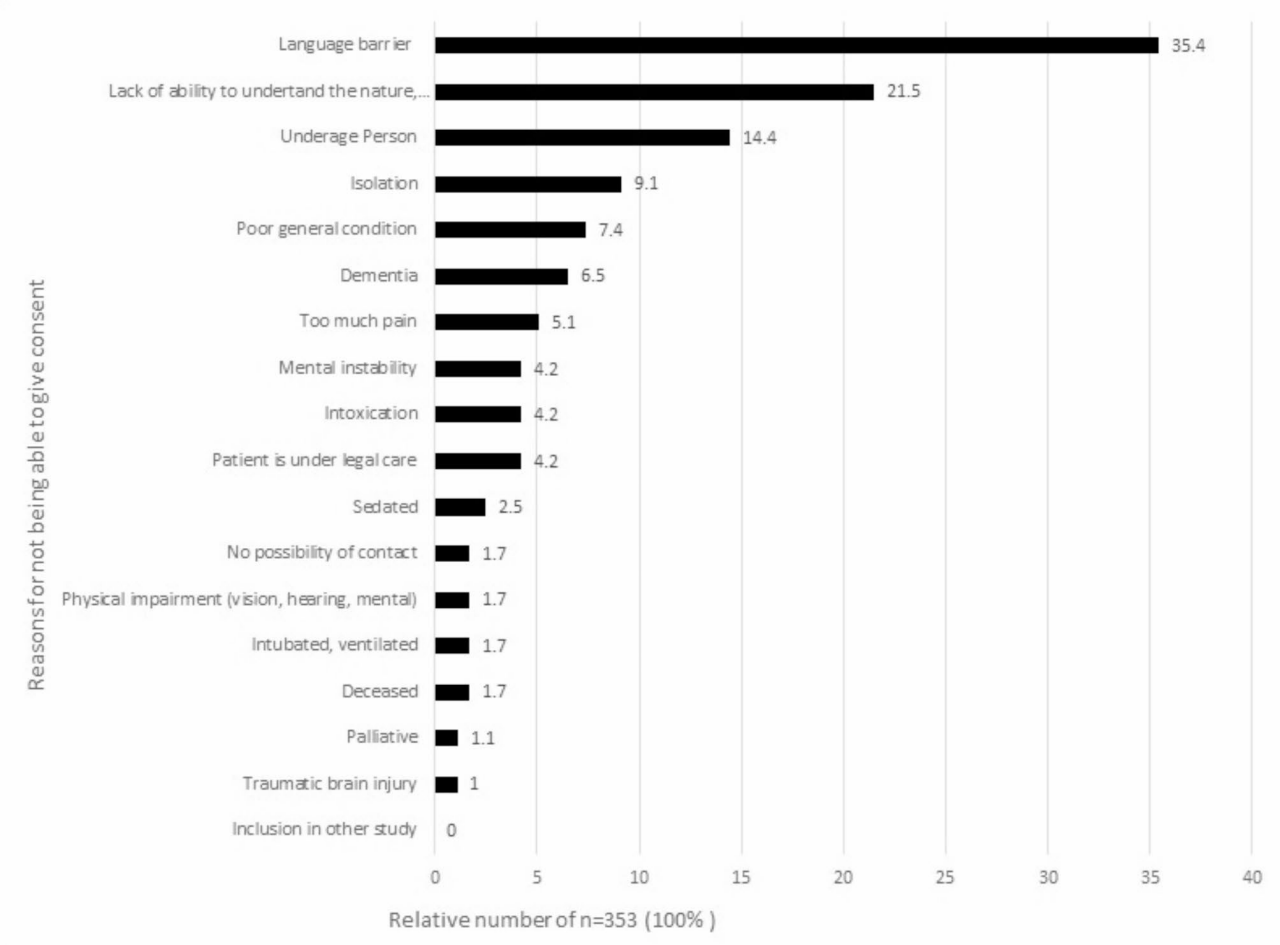
Reasons for not being able to consent in MII-BC in the ED setting (multiple answers possible). Shown are bar graphs for the relative proportions. Not specified *n* = 2

The reasons why consent could not be obtained are statements which were documented in the survey according to the assessment of the study nurse. The biggest hurdle to consent were language barriers with 35.4%, lack of ability to grasp the nature, significance and scope of the study (e.g. cognitive overload, attention problems) with 21.5%, lack of majority (over 18 years) with 14.4%, isolation^1^ (9.1%) and poor general health (7.4%, Fig. 3). Not included as a category in advance, but summarized by a relevant proportion of free text additions, have been palliative condition and physical impairment (vision, hearing, mental), intoxication and mental instability.

**Fig. 4:**
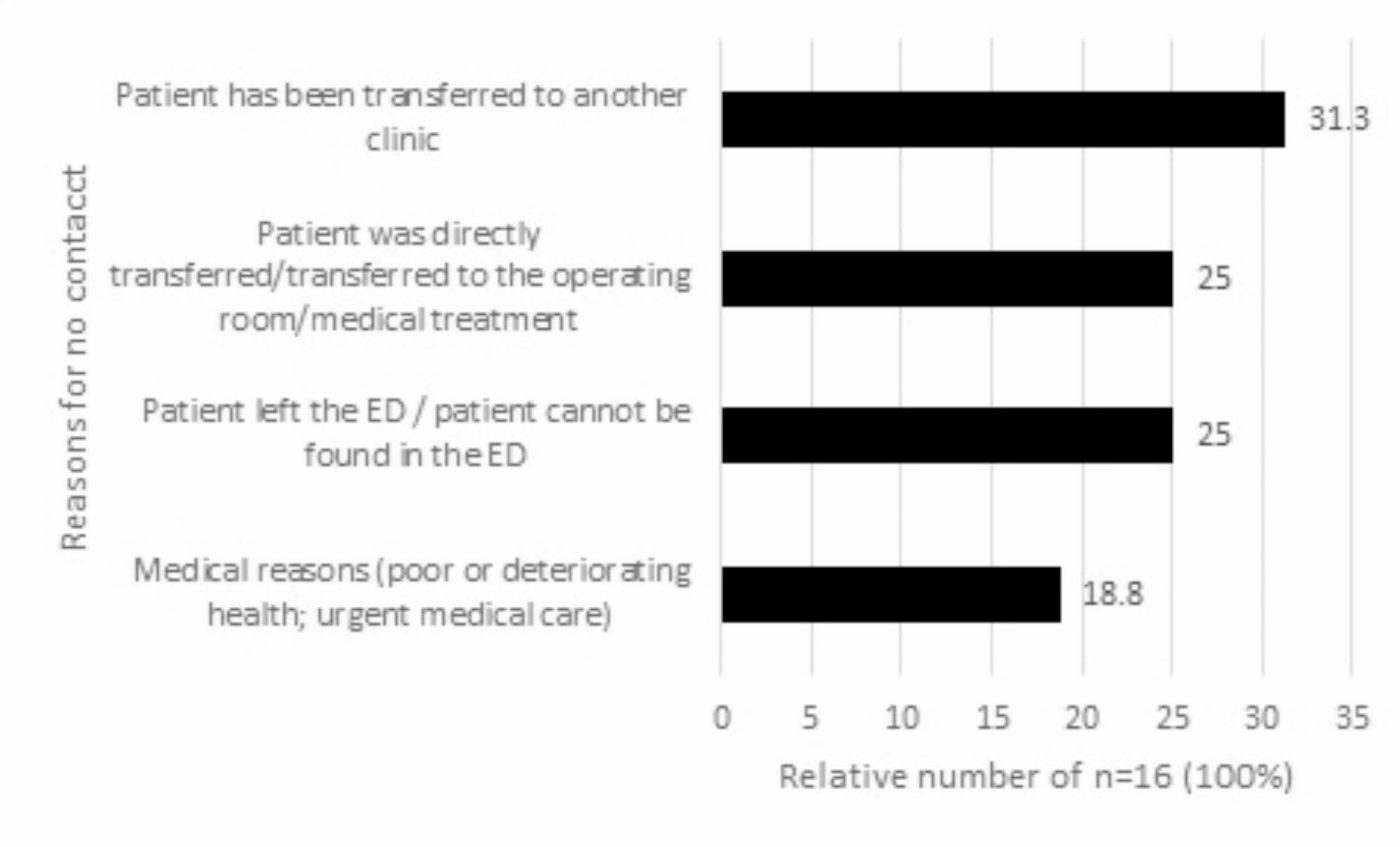
Reasons why the study nurse was unable to contact patient in the ED setting. Shown are bar graphs for the relative proportions. No Missings

18 patients (3.3%) capable of giving consent could not be contacted because they had already been transferred to a different facility or could not be contacted due to urgent treatment (Fig. 4).

**Fig. 5:**
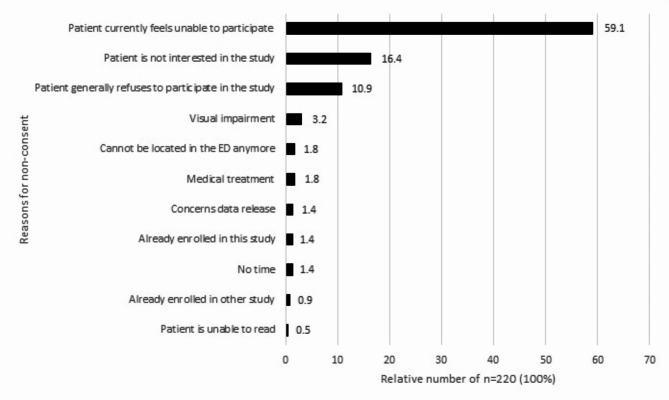
Reasons for non-consent in the MII-BC if the patient was contacted by the study nurse in respect of the selection of every fifth or 30th patient (*n* = 220). Shown are bar graphs for the relative proportions. No Missings

Of those 535 patients who were capable of giving consent and could be contacted by the study nurses, 313 patients consented to MII-BC (consent rate 27.5% total baseline population and 58.5% approached). The reasons why consent was refused were recorded by the study nurse in the survey (Supplement 3, Question 3a). Three categories were predefined and the rest were re-categorized during the data analysis by specifying “Other” which was further collected as a free text option. The most common reason for the 220 refusing patients was that they did not feel able to consent at this time (59.1%), 16.4% were not interested in this study and 10.9% generally refused to participate in any study (Fig. 5; Table 1 (Supplement 5).

**Table 1:** Sociodemographic characteristics of the consented participants in BC-ED. Answers were self-reported by the participants in the questionnaire at the end of the study procedure

Question	Category	Absolut counts	Relative proportion
Age in years shown as Median (25%-75% Quartile) (Missing *n* = 1)	43.0 (29.3–58.0)		
Gender	Female	86	38.2
	Male	133	59.1
	Other	1	0.4
	Prefer not to answer	2	0.9
	Missing	3	1.3
Assigned gender at birth	Female	86	38.2
	Male	129	57.3
	Other	0	0.0
	Prefer not to answer	7	3.1
	Missing	3	1.3
Country of birth	Germany	195	86.7
	EU	13	5.8
	Outside the EU	12	5.3
	Missing	5	2.2
Which language do you prefer? Free text field	German	178	79.1
	German combined with another language	9	4.0
	Other (Albanian, English, French, Indian, Italian, Polish, Romanian, Russian)	10	4.4
	Missing	28	12.4
How well do you consider yourself to speak German?	First language	179	79.6
	Fluently	25	11.1
	Good knowledge	8	3.6
	Prefer not to answer	2	0.9
	Missing	11	4.9
What is your highest school-leaving qualification?	Abitur, general higher education entrance qualification	88	39.1
	Advanced technical college entrance qualification, Qualification for entry to a senior technical school	29	12.9
	Intermediate school	44	19.6
	Secondary school/elementary school	40	17.8
	Finished school without qualification	2	0.9
	Other school-leaving qualification (e.g. obtained abroad)	6	2.7
	Prefer not to answer	8	3.6
	Missing	8	3.6
What is your highest educational qualification?	University	54	24.0
	University of applied sciences, engineering school	24	10.7
	Technical school (vocational or technical academy)	30	13.3
	Apprenticeship (vocational training)	70	31.1
	No qualification or still in vocational training	15	6.7
	Other educational qualification	7	3.1
	Prefer not to answer	13	5.8
	Missing	12	5.3
Are you currently…	Full-time employed	99	44.0
	Part-time employed	27	12.0
	Self-employed	12	5.3
	On parental leave / maternity leave	1	0.4
	Pupil and student	21	9.3
	Not employed	12	5.3
	Retired, pension, early retirement	46	20.4
	Prefer not to answer	6	2.7
	Missing	1	0.4
How many people live in your household, including you?	1	39	17.3
	2	90	40.0
	3	36	16.0
	4	26	11.6
	> 5	13	5.8
	Prefer not to answer	15	6.7
	Missing	6	2.7
How many are younger than 14 years?	0	137	60.9
	1	22	9.8
	2	18	8.0
	≥ 3	6	2.6
	Missing	42	18.7
What is the total monthly net income of your household?	Above 2500 Euro	93	41.3
	About 2500 Euro	16	7.1
	Below 2500 Euro	49	21.8
	Prefer not to answer	64	28.4
	Missing	3	1.3
Do you have a nursing care level?	1	4	1.8
	2	4	1.8
	3	4	1.8
	4	0	0.0
	5	1	0.4
	None	197	87.6
	Prefer not to answer	8	3.6
	Missing	7	3.1
What is your current marital status?	Single	93	41.3
	Divorced	13	5.8
	Married (living together)	96	42.7
	Married (living apart)	5	2.2
	Registered partnership (living together)	5	2.2
	Widowed	5	2.2
	Prefer not to answer	6	2.7
	Missing	2	0.9
How do you identify yourself?	Queer	3	1.3
	Lesbian	0	0.0
	Gay	4	1.8
	Bisexual	7	3.1
	Heterosexual	162	72.0
	Prefer not to answer	32	14.2
	Missing	17	7.6
Where do you currently live?	In an apartment or house (owned, rented or with relatives)	212	94.2
	Assisted living (e.g. retirement apartments, retirement homes, senior residences, senior-friendly living)	2	0.9
	Other	1	0.4
	Prefer not to answer	7	11
	Missing	3	1.3

### Study population– self reported characteristics

On average, patients were 43.0 years old (IQR 29.3–58.0) and 38.2% of the patients were female. 58.2% had an urgent triage level of category one to three, and 40.5% a less urgent triage level of category four or five. Language preference was German in 79.1%. 52% had a high-school diploma, 34.7% a university or university of applied sciences degree, 56.0% worked part or full time, 17.3% lived in a single household, 28.9% had a total of 2,500€ or less/month at their households’ disposal, 5.8% had nursing care level, 41.3% were single. More detailed information is shown in Table 1 (Supplement 5).

### Motivation to provide BC

**Fig. 6:**
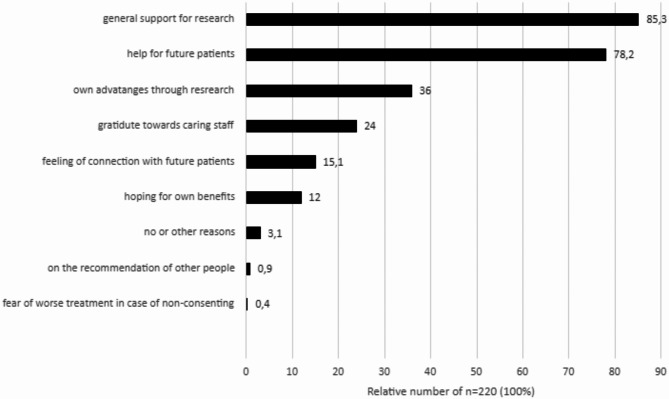
Reasons for motivation to consent in the MII-BC in the ED setting (multiple answers possible). Shown are bar graphs for the relative proportions. No Missings

Patients who consented the MII-BC have indicated general support for research (85.3%), followed by 78.2% expressing a desire to help future patients and 36.0% of the patients suspect own advantages from research (Fig. 6). In contrast, only one patient (0.4%) stated that consent would have been given due to fear of receiving worse treatment in case of non-consenting.

### Description of the MII-BC process

Regarding the consenting process, 80 interruptions (25.6% of consented patients) were documented with a median duration of 15 min (IQR 5.0–20.0) and 8 patients asked for reflection time (2.6% of the consented patients) with a median of 12.5 min (IQR 6.5–45.0). The consent process took 10 min (IQR 5–24) from the start of the patient information on MII-BC until consent was given.

In total there have been 20 study drop outs before completing the study registered (6.4% of the consented patients). 12 of these were due to the patients’ request for personal reasons (3.8% of consented patients), four due to medical reasons like deterioration of the health condition or urgent medical care without the possibility of continuing the conversation afterwards (1.3% of consented patients), and four times the patient could not be located again due to transfer or leaving the ED (1.3% of consented patients).

The consent process took place in 31.3% of cases while the patients were sitting in the waiting area, 31.0% of cases by telephone, in 25.3% of cases in the treatment or in a separate room, in 7.7% of cases on the ward, in 3% of cases lying in the waiting area.

### Recall of the content of the MII-BC

Table 2 provides an overview of the MII-BC modules to which consent was documented by the study nurse in the survey form, compared with the modules recalled and documented in the patients’ questionnaire. Most patients consented to the use of their clinical data (future data 90.2%, retrospective data 84.4%). Consent for the use of biomaterials was obtained from 65.3% for future data and 61.8% for retrospective data. Overall discrepancies between the survey form and the questionnaire, reflecting differences in the patients’ recall of which modules they consented to, ranged from 9.8% (‘Retrospective health insurance data’) and 16.4% (‘Current biomaterial’). Mostly this was due to not remembering the consent in distinctive modules by the patient, which is shown in Table 2 column “absolute discrepancies”.

**Table 2:** Absolute (numbers) and relative proportion (percents) of the different MII-BC modules transferred by the study nurse directly from the consent form and recalled by the patient through post-consent questioning. Reported are answers for and from 225 patients (100%). * absolute discrepancies: first number in brackets - entry in the study nurse survey form but module not checked in the questionnaire, second number in brackets module checked in the questionnaire but not in the survey form - numbers separated by “vs.”. MII-BC: broad consent form introduced by the Medical Informatics Initiative

	Study nurse survey form		Patient questionnaire		Discrepancies	
MII-BC module	Absolute	Relative	Absolute	Relative	Absolute (*)	Relative
Future clinical data	203	90.2	172	76.4	35 (33 vs. 2)	15.6
Retrospective clinical data	190	84.4	165	73.3	27 (26 vs. 1)	12.0
Retrospective health insurance data	166	73.8	146	64.9	22 (21 vs. 1)	9.8
Prospective health insurance data	169	75.1	146	64.9	25 (24 vs. 1)	11.1
Future biomaterial	147	65.3	126	56	37 (29 vs. 8)	16.4
Retrospective biomaterial	139	61.8	130	57.8	33 (21 vs. 12)	14.7
Recontacting further questions	173	76.9	150	66.7	33 (28 vs. 5)	14.7
Additional medical findings	175	77.8	144	64.4	35 (33 vs. 2)	15.6

### Understanding and satisfaction with the MII-BC process

Some results regarding the understanding of the MII-BC are submitted for publication by Eienbroeker et al. [18] along with results of participant observations in this study. 1.3% would prefer to be informed by a doctor and 3.6% reported not having enough time to think about consenting to MII-BC, when asked about rating the process. 89.8% felt sufficiently informed about the scientific use of the data and 85.7% felt sufficiently informed about the use of biomaterials. 94.0% also stated that they had enough time to think about the procedure. 91.1% felt well informed about the benefits and 82.2% about the risks. When asked in detail, 95.1% said they understood the content well, while 90.7% said they felt their concerns were well addressed. The results are presented in detail in the tabl of the Supplement (Supplement 5).

## Discussion

### Consent rate and motivation

In our study, we observed a consent rate of 58.8% in the MII-BC among the 535 patients in the ED which could be approached by the study nurse. This is a bit below the consent rate of other studies investigating the concept of BC for data collection and biomaterial donation, for example Ewing et al. found 70% [19]. This is due to the special population in any ED, where not all patients could be approached per se. In our study, the recruitment of ED patients provided a wide-ranging, non-preselected spectrum of disease, including both outpatient and inpatient cases, as well as varying degrees of disease severity.

The willingness and motivation to provide data and biomaterial is high. With regards to the consent for the individual modules of the MII-BC separately we found a consent rate of 90.2% for current clinical data, 84.4% for retrospective clinical data, 73.8% for retrospective health insurance data, 75.1% for prospective health insurance data, 65.3% for current biomaterial, 61.8% for retrospective biomaterial, 76.9% for re-contacting for further questions and 77.8% for information regarding additional medical findings (survey and questionnaires available *n* = 225). A review determined a 70% consent rate for the use of clinical data [20]. Consent for biomaterial donation was 93% in a study by Pillai et al. [21] while ours have been lower at 65.3%. Two studies by Richter et al. were able to determine an acceptance rate of 87% with regard to BC for biobanking [22] and over 90% in Dutch and German patients for secondary data. In the latter work, however, the large difference between the motivation for public research (92%) versus private research (14%) was explicitly pointed out [23]. The BC and “open data sharing” are not an obstacle to consent [24] and one of the most important motivations is trust in research [25–28] which is in line with our findings, where 85.3% of participants want to support research in general. A review by Kalkman shows that support for data donation is high, but there are concerns about misuse of the data. This is particularly true for commercial use and patients want to know what is happening to their data [29]. Two aspects suggesting that patients perceived the voluntary nature of consent are, firstly, the very low rate of response given for “fear of worse treatment in case of non-consenting” (0.4%) and secondly, the still low rate of response for “hoping for own benefits” (12.0%). We were able to show that patients gave their consent voluntarily and did not feel pressured to agree, nor did they do so with the expectation of better treatment which is a very important ethical aspect for general use of the BC [30]. Not applicable in our study, but in the context of ethical considerations important to mention is, that financial incentives exert a certain pressure on the patient to give consent. Voluntariness due to monetary incentives and thus possibly involuntary participation of economically disadvantaged patients is discussed by Yearby et al. [31], as well as by Chan et al. [32].

### Reasons for not being able to consent in the ED setting

This is the first study to assess selection bias regarding MII-BC in the ED setting. The biggest obstacle for being eligible for inclusion was the language barrier, which amounted to 35.4% in our study. Since ethically and legally a basic understanding of language is a fundamental requirement for informed consent, the language barrier is one of the sticking points to solve. Zeeshan et al. showed that patient recall depends on understanding the language of the informed consent [33]. Ngwenya et al. showed that most patients understanding is good when communication takes place at the appropriate language level with regard to the socioeconomic status as well [34]. The MII developed recently further information material about the MII-BC and consent forms are also available in English, Turkish, Arabic and simple language [11]. If those versions in different languages and language levels will be implemented successfully, this could increase inclusion of these not yet well reached patient groups in future studies. For the implementation of this study, it was important to obtain all regulatory requirements for the use of the same version of the BC (version 1.7.2). Some of the versions in the different languages and in plain language only appeared in the course of the study. Furthermore, the study nurse must also be linguistically competent to accompany this offer.

Other leading reasons for not being capable for giving consent were insufficient cognitive ability, which was subjectively assessed by the study nurse, and being underage. Versions of the MII-BC documents were also made available for this patient groups: There are newer versions for minors in two age groups (7–11 and 12–17 years) and their legal guardians [11].

Patients’ reasons for inability to give consent in the ED due to their current medical condition were because of isolation, poor general condition, excessive pain, intoxication of various origins, sedation, intubation or ventilation, deceased or traumatic brain injury. These reasons totalled 32.8%. Other reasons were dementia, care, mentally unstable condition and physical limitations. Our findings are consistent with the theoretical work by Furyk et al., who addressed the limitations of consent in the ED in general, taking into account ethical, logistical and regulatory constraints, and on the other hand emphasised the importance of reaching this heterogeneous clientele of patients who differ in their severity of treatment [35]. Chan and colleagues also discussed the complexity of obtaining consent from patients with severely impaired conditions in the ED. They highlighted the ethical challenge of justifying inclusion in a study but on the other hand addressing the research question well in terms of content [32]. Also Adamis et al. point out the difficulty of reaching patients with delirium for studies, which limits the generalisability of the results accordingly [36]. Due to ethical issues and the requirement to provide truly informed consented patients, most of those conditions can most likely not be addressed by future studies. However, our study also revealed the need to consider options for patients with visual impairment in future studies and also highlighted the necessity of facilitating consent by the legal carer in the context of MII-BC.

### Understanding and recalling the content of the MII-BC and possible strategies to improve

The patients’ self-reported general understanding in our study was 95.1%. With regard to the recalled modules in detail, the documentation of the study nurse according to the consent form was not always congruent with what the patient recalled in the questionnaire. The deviations for the individual modules varied between 9.8% and 16.4%, whereby the information in the patient questionnaire was mostly missing, although consent was originally given for the respective module. On the one hand, this suggests a reduced recall of the content of the consent, but at the same time this may be closely related to general understanding. This goes in line with Li et al. who showed that recall was also reduced in parents who were informed about emergency surgery for their children [37]. Eichner and colleagues showed a relatively high understanding of patients’ rights, but relatively low understanding of the basic content of the study [38, 39]. A review by Tam et al. showed a variation between 52.1% and 75.8% in the understanding of individual components of informed consent in clinical trials [40]. Also, the study by Hajivassiliou et al. showed a generally higher level of satisfaction with the information process as in our study, but a relatively low level of understanding. In this study, the term “informative-patient centered care” was defined and education was identified as an important factor [41].

In our study, a reflection period was only requested eight times and patients generally stated that they had enough time available. In the recall of the consent content patients showed memory gaps, this justifies future efforts to increase understanding to achieve a real shared medical decision-making process [42]. There are some ideas on how to improve the understanding and memorisation of consent. In a review, Nishimura et al. found that understanding is improved by endeavoring to provide as much detail as possible and offering plenty of opportunity for discussion [43]. Flory et al. postulated that a one-to-one discussion with trained study staff was effective in improving understanding. Further increased use of multimedia, “easy-to-read” and information tailored to the individual needs of patients is being discussed to increase understanding [44]. Also key information should be emphasised [45]. New communication strategies to increase understanding are also discussed by Kadam et al. The documentation must be simplified and the “teach back” method is suggested, in which the patient summarises in their own words what they have understood [46]. Most of these approaches are certainly too complex in the lively setting of the ED, but approaches such as “easy-to-read” materials and emphasising keywords are possible to implement here too. In the ED setting, but also beyond, it has been shown that the study nurse should be very well trained and familiar with the challenges of the ED setting.

### Strengths and limitations of the study

This study was the first to address the potential feasibility of MII-BC in the ED at four sites in Germany. Extensive structured information on selection bias and resource use in the context of consent was collected, as well as patient-reported information on voluntariness, understanding, satisfaction and recall.

As mentioned in the method section the project duration of CODEX+ (beginning of 2022 to end of 2022) was very short, many decisions regarding the implementation of the project were made with a focus on the feasibility under these circumstances. A limitation of this feasibility study was the lack of multilingual questionnaires and informed consent processes, which restricted the inclusion of non-German-speaking patients and potentially reduced the diversity of the study population. This constraint arose due to the short implementation timeframe, the late availability of multilingual materials, and the inability of study nurses to conduct informed consent discussions in multiple languages.

To meet the regulatory and structural requirements and resources available, different strategies had to be implemented at the study sites (“real” versus “hypothetical consenting”, direct every fifth patient during working hours of the study nurse versus indirect contacting every thirtieth patient 24/7, with and without biomaterial, delayed consenting in Freiburg). Detailed results of the delayed consent process are published elsewhere [10]. This could influence the comparability of the results at the study sites. The hypothetical process for example was also somehow confusing for the patients as they had to consent twice - once to participate in general to the BC-ED study and once to consent in the MII-BC. Consenting at that point of the study led potentially to a selection bias of participating patients.

Further it was not possible to carry out extensive piloting after developing the entire study procedure and passing the regular requirements. There was only a very short test of the survey and questionnaire (five patients at each study site), as well as establishing consistent documentation. This could have reduced the proportion of missing questionnaires (88 missing questionnaires) and also increased the completeness of missing values within the questionnaires (forgotten or knowingly not answered). To this end, an intensive weekly exchange took place between the study sites in order to discuss the different constellations together and try to harmonise the procedures and documentation in the best possible way. Even the assessment of the study nurse if the patient is capable of giving consent was often not trivial and might have been carried out differently elsewhere. This was the case, for example, with regard to the cognitive capability of patients or the assessment of their general medical condition. Even during the consent process, the study nurse sometimes had to adjust their initial assessment and cancelled the process accordingly. The rate of patients not capable of giving consent was between 36.3% and 66.7% at the different study sites. To harmonise those quite subjective and often sensitive aspects of studies between different study sites is always a challenge and should be addressed with extensive exchange between participating study sites.

Finally, we would like to point out that for future studies, the involvement of patients and the public should already be considered in the brainstorming for project ideas, conception as well as evaluation phases of studies. Unfortunately, we were not able to implement this in our project.

## Conclusion

In conclusion, our study addresses the challenges surrounding the implementation of BC, particularly within the dynamic and demanding ED setting for the first time. Among all 1,138 ED patients in this study, 535 could be approached by the study nurse and were asked for broad consent. Of these the consent rate was 27.5% of the total baseline population and 58.5% of those approached, reflecting comparable rates to previous studies investigating BC for data collection and biomaterial donation so far in other settings. Despite encountering obstacles such as language barriers, cognitive limitations, and medical conditions rendering patients unable to provide consent, our findings indicate a considerable level of willingness and motivation among patients to contribute to medical research.

The reasons for not being able to consent, underscore the need for strategies to enhance inclusivity in consent processes, such as providing consent documents in multiple languages and adapting materials for individuals with cognitive impairments or medical complexities. Currently, existing inclusion options for consenting continue to lead to a healthier and in some respects to a still selected patient population of younger, less severely ill patients who would not be representative for the whole ED population.

Furthermore, our study sheds light on the importance of understanding and recall in the consent process. While patients generally reported a high level of understanding and satisfaction with the information provided, discrepancies between documented consent and patient recall indicate the need for continued efforts to improve comprehension and retention of consent details. Strategies such as one-on-one discussions, multimedia presentations, and simplified documentation may enhance patient understanding and recall, thereby promoting informed decision-making in research participation.

## Electronic supplementary material

Below is the link to the electronic supplementary material.

Supplementary Material 1

Supplementary Material 2

Supplementary Material 3

Supplementary Material 4

Supplementary Material 5

## Data Availability

Data is provided within the manuscript or supplementary information files.

## Footnotes

1 The term “isolation” referred to patients who were in isolation rooms due to the risk of infection. In the interests of health safety for the study nurses, consent was not sought here.
