# Supplementary material for: Broad consent in the emergency department: a cross sectional study
Source: Arch Public Health. 2025 Feb 18;83:44. doi: 10.1186/s13690-025-01529-z (PMC11834566; doi:10.1186/s13690-025-01529-z)
Supplement: Supplementary file 1 — Supplementary Material 1 [file 13690_2025_1529_MOESM1_ESM.pdf]

Arbeitsgruppe Consent
Mustertext Patienteneinwilligung

(Stand 16.03.2021)

Version 1.7.2

bestehend aus Patienteninformation und -einwilligung

Patienteninformation

zur Nutzung von Patientendaten [*falls zutreffend:* Krankenkassendaten und Biomaterialien (Gewebe und Körperflüssigkeiten)] für medizinische Forschungszwecke

Sehr geehrte Patientin, sehr geehrter Patient!

Sie werden gegenwärtig an [unserem/unserer *Name der behandelnden Einrichtung*] zur Diagnosestellung oder Therapie ärztlich behandelt. Im Rahmen Ihrer Behandlung werden von Ihnen Patientendaten erhoben [*falls zutreffend*: und eventuell auch Biomaterialien (Gewebe & Körperflüssigkeiten) gewonnen, z.B. im Rahmen von Blutentnahmen, Biopsien oder operativen Eingriffen]. Diese Patientendaten [*falls zutreffend:* und Biomaterialien] können für die medizinische Forschung von erheblichem Wert sein.

Medizinische Forschung ist notwendig, um die Früherkennung, Behandlung und Vorbeugung von Krankheiten laufend zu verbessern; dazu können Erkenntnisse, die wir aus Ihren Patientendaten und Biomaterialien gewinnen möglicherweise sehr viel beitragen. Wir möchten Sie daher bitten, uns Ihre Patientendaten [*falls zutreffend:* und Biomaterialien] für medizinische Forschungszwecke zur Verfügung zu stellen. Ihre Patientendaten sollen dabei in einer Datenbank gesammelt werden, die durch [*Träger der Datenbank*] betrieben wird. [*Falls zutreffend:* Die qualitätskontrollierte Langzeit-Lagerung der von Ihnen zur Verfügung gestellten Biomaterialien erfolgt in Biobanken bzw. Archiven [*der/des Träger der Biobank(en) bzw. Archive*].

Ihre Einwilligung ist freiwillig. Wenn Sie sich nicht beteiligen möchten oder Ihre Einwilligung später widerrufen möchten, erwachsen Ihnen daraus keine Nachteile.

Wenn Sie mit der nachfolgend beschriebenen Art und langfristigen Dauer der Nutzung nicht in vollem Umfang einverstanden sind oder Ihre Rückfragen nicht alle zufriedenstellend beantwortet wurden, sollten Sie Ihre Einwilligung nicht erteilen.

1. Erhebung, Verarbeitung und wissenschaftliche Nutzung Ihrer Patientendaten

1.1 Welche Ziele verfolgen wir?

Ihre Patientendaten sollen für die medizinische Forschung zur Verfügung gestellt werden. Medizinische Forschung dient ausschließlich dazu, die Erkennung, Behandlung und Vorbeugung von Krankheiten zu verbessern; Ihre Patientendaten werden nicht für die Entwicklung biologischer Waffen oder diskriminierende Forschungsziele verwendet. **Ebenso ist es nicht Ziel dieser Forschung, bei Ihnen eine Diagnose zu erstellen oder Ihre konkrete Behandlung zu beeinflussen.**

Ihre Patientendaten sollen im Sinne eines breiten Nutzens für die Allgemeinheit für viele verschiedene medizinische Forschungszwecke verwendet werden. Zum jetzigen Zeitpunkt können dabei noch nicht alle zukünftigen medizinischen Forschungsinhalte beschrieben werden; diese können sich sowohl auf ganze Krankheitsgebiete (z.B. Krebsleiden, Herz-Kreislauf-Erkrankungen, Erkrankungen des Gehirns) als auch auf heute zum Teil noch unbekannte einzelne Krankheiten und Veränderungen in der Erbsubstanz beziehen. Es kann also sein, dass Ihre Patientendaten für Forschungsfragen verwendet werden, die wir heute noch gar nicht absehen können. Dazu sollen Ihre Patientendaten [*falls zutreffend:* und Biomaterialien] **für 30 Jahre ab dem Zeitpunkt Ihrer Einwilligung gespeichert** [*falls zutreffend für Biomaterialien:* **und gelagert**] werden, wenn Sie nicht vorher widerrufen haben. In besonderen Fällen können Daten [*falls zutreffend:* und Biomaterialien] auch über diesen Zeitpunkt hinaus von erheblicher Bedeutung für die Wissenschaft sein. In diesen Fällen würden wir in Abstimmung mit den zuständigen Datenschutzaufsichtsbehörden und einer unabhängigen Ethikkommission klären, ob auch eine weitergehende Nutzung Ihrer Daten [*falls zutreffend:* und Biomaterialien] möglich ist.

Patientendaten

Patientendaten sind alle Informationen zu Ihrer Person, die anlässlich Ihrer Untersuchung und Behandlung genutzt werden. Beispiele für Patientendaten sind: Daten aus Arztbriefen, Ihre Krankengeschichte oder Befunde und Daten aus medizinischen Untersuchungen wie Blutdruckmessungen oder Röntgenbildern; ebenso zählen die Ergebnisse von Laboruntersuchungen dazu, einschließlich Untersuchungen Ihrer Erbsubstanz (z.B. auf angeborene genetisch bedingte Erkrankungen oder erworbene genetische Veränderungen, unter anderem auch von Tumoren).

[*Falls zutreffend:*

Möglicherweise wurden Sie schon früher einmal bei uns behandelt. Diese Behandlungen können unter Umständen schon sehr lange zurückliegen und auch andere Erkrankungen betreffen. Wenn es solche Behandlungen gab, wurden von Ihnen auch damals schon Patientendaten erhoben, die für die medizinische Forschung ebenfalls von erheblichem Wert sein können. In der Einwilligungserklärung können Sie uns auch die Nutzung Ihrer früheren Patientendaten erlauben. Auch diese Patientendaten können dann **für 30 Jahre ab dem Zeitpunkt Ihrer Einwilligung gespeichert und wissenschaftlich genutzt werden**, wenn Sie nicht vorher widerrufen haben.

***Ende Modul Retrospektive Daten – Abschnitt „Patientendaten“*]**

1.2 Wie werden Ihre Patientendaten wissenschaftlich genutzt?

Ihre Patientendaten können Universitäten, Forschungsinstituten und forschenden Unternehmen auf Antrag für medizinische Forschungszwecke zur Verfügung gestellt werden. Diese Daten dürfen vom Empfänger nur zu dem vorbestimmten und beantragten Forschungszweck genutzt und nicht zu anderen Zwecken weitergegeben werden. Ihre Patientendaten [*falls zutreffend:* und gespendeten Biomaterialien] werden ausschließlich für wissenschaftliche Zwecke genutzt; sie werden nicht verkauft. [*Die/Das Name der behandelnden Einrichtung*] kann aber für die Bereitstellung qualitätskontrollierter Daten von den jeweiligen Nutzern eine angemessene Aufwandsentschädigung erheben.

Die Zulässigkeit jedes einzelnen Forschungsvorhabens mit Ihren Patientendaten [*falls zutreffend:* und Biomaterialien] wird vorab von einer unabhängigen Ethikkommission geprüft und erfordert deren zustimmende Bewertung.

Wissenschaftliche Veröffentlichungen von Ergebnissen erfolgen ausschließlich **anonymisiert**, also in einer Form, die keine Rückschlüsse auf Ihre Person zulässt. [*Falls genetische Untersuchungen vorgesehen sind:* Das gilt insbesondere auch für genetische Informationen. Möglich ist allerdings eine Aufnahme Ihrer genetischen Daten bis hin zur gesamten Erbsubstanz (Genom) in besonders geschützte wissenschaftliche Datenbanken, die für die Allgemeinheit nicht zugänglich sind.]

Anonymisierung

Bei der Anonymisierung werden Ihre Daten so verändert, dass sie Ihrer Person nicht mehr oder nur mit einem unverhältnismäßig großen technischen Aufwand zugeordnet werden können.

Ihre Patientendaten [*falls zutreffend:* und Daten aus der Analyse Ihrer Biomaterialien] können auch mit Ihren Daten aus Datenbanken anderer Forschungspartner (z.B. anderer Krankenhäuser, Institute oder Register) zusammengeführt werden. Voraussetzung dafür ist, dass Sie dieser Nutzung auch bei den entsprechenden Forschungspartnern zugestimmt haben.

1.3 Wer hat Zugang zu Ihren Patientendaten und wie werden diese geschützt?

Alle unmittelbar Ihre Person identifizierenden Daten (Name, Geburtsdatum, Anschrift etc.) werden durch eine Zeichenkombination ersetzt (Codierung). Dieses interne Kennzeichen sowie Ihre damit verbundenen Patientendaten [*falls zutreffend:* und Biomaterialien] können dann nicht mehr direkt Ihrer Person zugeordnet werden. Der Zusammenhang dieses internen Kennzeichens mit den Sie direkt identifizierenden Daten wird von einer unabhängigen internen Stelle oder insbesondere im Falle einer einrichtungsübergreifenden Zusammenführung von Daten von einer unabhängigen externen Treuhandstelle

[*auf Website verweisen, die diese Stelle(n) aufführt*] verwaltet. Ohne die Mitwirkung dieser Stelle können die für die medizinische Forschung bereitgestellten Patientendaten nicht oder nur mit unverhältnismäßig hohem technischem Aufwand zu Ihrer Person zurückverfolgt werden. Vor einer Weitergabe Ihrer Daten [*falls zutreffend:* und Biomaterialien] an Forscher außerhalb Ihrer behandelnden Einrichtung erfolgt zudem eine weitere Ersetzung des internen Kennzeichens durch eine neue Zeichenkombination.

Codierung

Bei der Erfassung von Patientendaten werden auch Informationen wie Ihr Name und Ihr Geburts-datum erfasst. Mit solchen Informationen kann leicht auf Sie persönlich geschlossen werden. Diese Informationen werden durch eine Kombination von Zeichen ersetzt. Auf diese Weise wird eine einfache Rückverfolgung zu Ihrer Person ausgeschlossen. Eine Rückverfolgung zu Ihrer Person erfolgt nur, wenn Ihre Patientendaten durch zusätzliche Informationen über Sie ergänzt werden sollen oder um erneut mit Ihnen in Kontakt zu treten (siehe unten Punkt 4).

Daten, die Ihre Person identifizieren, werden außer in von Ihnen erlaubten oder gesetzlich geregelten Fällen niemals an Forscher oder sonstige Dritte weitergegeben, insbesondere nicht an Versicherungsunternehmen oder Arbeitgeber.

Ihre Einwilligung umfasst auch die Möglichkeit, Ihre Patientendaten [*falls zutreffend:* und Biomaterialien] zu den genannten Zwecken an Empfänger in Staaten der Europäischen Union oder des Europäischen Wirtschaftsraums oder in weitere Länder, bei denen die Europäische Kommission ein angemessenes Datenschutzniveau festgestellt hat, zu übermitteln. [*falls ohne Drittstaatenmodul:* **Eine Übermittlung in andere Länder, in denen kein angemessenes Datenschutzniveau festgestellt wurde, ist ausgeschlossen.**]

[*Falls zutreffend:*

Eine Übermittlung in andere Länder, in denen kein angemessenes Datenschutzniveau festgestellt wurde, ist nur möglich, wenn Sie dem in der Einwilligungserklärung gesondert zustimmen. Diese Länder haben möglicherweise ein niedrigeres Datenschutzniveau als die EU. [Die/Das *Name der behandelnden Einrichtung*] sichert zu, auch in diesen Fällen für eine vertragliche Verpflichtung der Forschungspartner zur Einhaltung des EU-Datenschutz-Niveaus zu sorgen, soweit dies rechtlich möglich ist. Dennoch besteht das Risiko, dass staatliche oder private Stellen auf Ihre Patientendaten [*falls zutreffend:* und Biomaterialien] zugreifen, obwohl dies nach dem europäischen Datenschutzrecht nicht zulässig wäre. Beispielsweise kann in den USA ein Zugriff durch Geheimdienste auch ohne richterlichen Beschluss erfolgen. Zudem kann es sein, dass Ihnen dort weniger oder schlechter durchsetzbare Betroffenenrechte zustehen und es keine unabhängige Aufsichtsbehörde gibt, die Sie bei der Wahrnehmung ihrer Rechte unterstützen könnte.

***Ende Drittstaatenmodul*]**

Unter der Adresse www.medizininformatik-initiative.de/datennutzung können Sie jederzeit sehen, welche Studien mit Ihren oder den Patientendaten [*falls zutreffend:* und Biomaterialien] anderer Patienten durchgeführt werden. Zudem finden Sie unter dieser Adresse eine Möglichkeit, sich für einen E-Mail-Verteiler zu registrieren, der Sie per E-Mail über alle neuen Studien mindestens eine Woche vor einer Datennutzung informiert.

1.4 Welche Risiken sind mit der Nutzung Ihrer Patientendaten verbunden?

Bei jeder Erhebung, Speicherung und Übermittlung von Daten im Rahmen von Forschungsprojekten mit Patientendaten [*falls zutreffend:* und Daten aus der Analyse Ihrer Biomaterialien] besteht durch das Hinzuziehen weiterer Informationen, z.B. aus dem Internet oder sozialen Netzwerken, das Restrisiko einer Rückverfolgbarkeit zu Ihrer Person. Das ist insbesondere dann der Fall, wenn Sie selbst genetische oder andere Gesundheitsdaten, z.B. zur Ahnenforschung im Internet, veröffentlichen.

Grundsätzlich erhöht ist das Risiko einer Rückverfolgbarkeit bei genetischen Patientendaten. Die Erbinformation eines Menschen ist in der Regel eindeutig auf eine Person bezogen, also auch auf Sie.

Zudem kann aus Ihren genetischen Daten in manchen Fällen auch auf Eigenschaften Ihrer Verwandten geschlossen werden.

Sollten Ihre Daten trotz umfangreicher technischer und organisatorischer Schutzmaßnahmen in unbefugte Hände fallen und dann trotz fehlender Namensangaben ein Rückbezug zu Ihrer Person hergestellt werden, so kann eine diskriminierende oder anderweitig für Sie und ggf. auch nahe Verwandte schädliche Nutzung der Daten nicht ausgeschlossen werden.

1.5 Welcher Nutzen ergibt sich für Sie persönlich?

Persönlich können Sie für Ihre Gesundheit im Regelfall keinen unmittelbaren Vorteil oder Nutzen aus der wissenschaftlichen Nutzung Ihrer Patientendaten [*falls zutreffend:* und Biomaterialien] erwarten. Auf Ihre aktuelle medizinische Behandlung wird Ihre Einwilligung somit keine Auswirkung haben. Sollte aus der Forschung ein kommerzieller Nutzen, z.B. durch Entwicklung neuer Arzneimittel oder Diagnoseverfahren, erzielt werden, werden Sie daran nicht beteiligt.

Es ist jedoch im Einzelfall möglich, dass ein Auswertungsergebnis für Ihre Gesundheit von so erheblicher Bedeutung ist, dass ein Arzt oder Forscher eine Kontaktaufnahme als dringend notwendig erachtet. Das ist insbesondere der Fall, wenn sich daraus ein dringender Verdacht auf eine schwerwiegende, bisher möglicherweise nicht erkannte Krankheit ergibt, die behandelt oder deren Ausbruch verhindert werden könnte.

Darüber hinaus können sich weitere Analyseergebnisse ergeben, die möglicherweise für Ihre Gesundheit relevant sind (Zusatzbefunde) und über die wir Sie informieren möchten. Sie können entscheiden, ob wir Sie in diesem Zusammenhang kontaktieren dürfen. Beachten Sie dabei, dass Sie Gesundheitsinformationen, die Sie durch eine solche Rückmeldung erhalten, unter Umständen bei anderen Stellen (z.B. vor Abschluss einer Kranken- oder Lebensversicherung) offenbaren müssen und dadurch Nachteile erleiden könnten. Da für die medizinische Forschung eventuell auch Informationen aus Ihrer Erbsubstanz genutzt werden sollen, kann sich das auch auf Ihre genetische Veranlagung für bestimmte Erkrankungen beziehen. Weitere Informationen zu genetischen Daten finden Sie unter www.vernetzen-forschen-heilen.de/genetische-daten.

Informationen aus Ihrer Erbsubstanz können auch Bedeutung für Ihre Familienangehörigen und die Familienplanung haben. Sie können Ihre Entscheidung für oder gegen diese Möglichkeit zur Rückmeldung jederzeit durch Mitteilung an uns ändern.

1.6 Welcher Nutzen ergibt sich für unsere Gesellschaft?

Medizinisch-wissenschaftliche Forschungsvorhaben zielen auf eine Verbesserung unseres Verständnisses der Krankheitsentstehung und der Diagnosestellung, und auf dieser Basis auf die Neuentwicklung von verbesserten Präventions-, Versorgungs- und Behandlungsansätzen. Weitere Informationen über unsere Aktivitäten finden Sie unter [*Homepage*].

[*Falls zutreffend:*

2. Übertragung und wissenschaftliche Nutzung von Krankenkassendaten

Krankenkassendaten

*Bei Ihrer Behandlung [*in der/im Name der behandelnden Einrichtung*] werden nur Daten erhoben, die im unmittelbaren Behandlungszusammenhang benötigt werden. Für viele wissenschaftliche Fragestellungen reichen diese „Momentaufnahmen“ aber meist nicht aus. Um ein umfassenderes Bild von Ihrem Gesundheitszustand zu erhalten, würden wir z.B. gerne auch Ihre Patientendaten aus der ambulanten Versorgung nutzen. Über diese Informationen verfügt Ihre Krankenkasse.*

Wir bitten Sie darum, dass wir von Ihnen auch Daten, z.B. über vorangegangene und nachfolgende Arztkontakte bei ambulanten Haus- und Fachärzten sowie ggf. von weiteren Krankenhausaufenthalten und Arzneimittel-Verordnungen anfordern und wissenschaftlich nutzen dürfen. Unter Punkt 2 in der Einwilligungserklärung können Sie uns dazu ermächtigen, die entsprechenden Daten bei Ihrer

Krankenkasse anzufordern. Die Krankenkassen erhalten von uns aber keinerlei Forschungsergebnisse, die Ihnen persönlich zugeordnet werden könnten Damit wird Ihnen auch kein Nachteil durch die Nutzung Ihrer Krankenkassen-Daten entstehen.

***Ende Modul Kassendaten*]**

[*Falls zutreffend:*

3. Gewinnung, Lagerung und wissenschaftliche Nutzung von Biomaterialien (Gewebe und Körperflüssigkeiten)

3.1 Was sind Biomaterialien?

Biomaterialien
*Unter Biomaterialien versteht man Gewebeproben und/oder Körperflüssigkeiten, die Ihnen zur Diagnosestellung oder Therapie entnommen wurden und die nach Abschluss der Untersuchungen nicht mehr benötigt werden (Restmaterialien). Dabei kann es sich z.B. um Blut, Urin, Stuhl, Speichel, Hirnwasser oder um Gewebe handeln, das z.B. im Rahmen einer Operation oder bei einer Biopsie entnommen wurde. Diese Restmaterialien können für die medizinische Forschung nützlich sein und sollen dafür in Biobanken bzw. Klinik- oder Institutsarchiven aufbewahrt werden. [*Falls zutreffend:* Darüber hinaus können Sie bei einer Routine-Blutentnahme bzw. ohnehin geplanten Punktion auch zusätzliche Proben (z.B. eine begrenzte zusätzliche Blutmenge) für medizinische Forschungszwecke spenden (siehe unten Punkt 3.2).]*

[*Falls zutreffend:*

Möglicherweise wurden Sie schon früher einmal bei uns behandelt. Diese Behandlungen können unter Umständen schon sehr lange zurückliegen und auch andere Erkrankungen betreffen. Wenn es solche Behandlungen gab, wurden Ihnen auch damals vielleicht schon Biomaterialien entnommen, die für die medizinische Forschung ebenfalls von erheblichem Wert sein können. In der Einwilligungserklärung können Sie uns auch die Nutzung der früher entnommenen Biomaterialien erlauben. Auch diese Biomaterialien können dann **für 30 Jahre ab dem Zeitpunkt Ihrer Einwilligung gelagert und wissenschaftlich genutzt werden**, wenn Sie nicht vorher widerrufen haben.

***Ende Modul Retrospektive Daten – Abschnitt „Biomaterialien"*]**

3.2 Wie werden Ihre Biomaterialien wissenschaftlich genutzt und vor Missbrauch geschützt?

Für den Umgang mit Ihren Biomaterialien und den daraus gewonnenen Daten sowie für die damit verbundenen Ziele und Risiken gelten dieselben Regeln und Grundsätze, die oben zu den Patientendaten erläutert worden sind. Die Einzelheiten ergeben sich aus den Abschnitten 1.1 – 1.6 dieser Patienteninformation. In Biomaterialien kann Ihre Erbsubstanz in Form genetischer Daten enthalten sein. Insofern sind insbesondere die unter 1.4 beschriebenen Risiken für genetische Daten zu beachten. Hierzu zählt auch ein erhöhtes Risiko einer Rückverfolgbarkeit Ihrer Person anhand dieser Daten.

Ihre Biomaterialien sollen für verschiedene medizinische Forschungszwecke verfügbar sein. Dazu werden diese in einer Biobank bzw. einem Archiv [*der/des Träger der Biobank(en) bzw. Archive*] aufbewahrt und können auf Antrag auch an andere Forschungspartner weitergegeben werden.

Zu den Forschungsvorhaben mit Ihren Biomaterialien können auch genetische Untersuchungen zählen, einschließlich Untersuchungen Ihrer Erbsubstanz, z.B. auf angeborene genetisch bedingte Erkrankungen oder erworbene genetische Veränderungen, unter anderem auch von Tumoren. Dies kann unter Umständen auch eine Untersuchung Ihrer gesamten Erbsubstanz (Genom) umfassen.

[*Falls zutreffend:* Für die Forschung kann es sehr hilfreich sein, bei einer sowieso stattfindenden Routine-Blutentnahme oder -Punktion etwas mehr Biomaterial zu entnehmen, als für die Unterstützung Ihrer Behandlung nötig ist. Diese zusätzliche Entnahme wird nur vorgenommen, wenn Sie dem gesondert in der Einwilligungserklärung zustimmen. Zudem ist diese zusätzliche Entnahme zu Ihrem Schutz begrenzt. [*Entweder:* Innerhalb von [*lokal abgestimmtes Intervall einsetzen*] *oder:* Pro Materialgewinnung]

dürfen nach Maßgabe Ihres behandelnden Arztes insgesamt höchstens [*lokal abgestimmtes Maximum einsetzen*] Blut oder Punktionsflüssigkeit (ca. [*lokal abgestimmter Wert*] Teelöffel voll), bei Hirnwasser bis zu [*lokal abgestimmtes Maximum einsetzen*] (ca. [*lokal abgestimmter Wert*] Teelöffel voll) zusätzlich für Forschungszwecke entnommen werden. Entnahmemengen oberhalb dieser Grenzen erfordern eine gesonderte Aufklärung und Einwilligung.]

3.3 Wer erhält Eigentum an Ihren Biomaterialien?

Mit der Einwilligung zur Gewinnung, Lagerung und wissenschaftlichen Nutzung Ihrer Biomaterialien wird gleichzeitig das Eigentum an den Biomaterialien an [*den/die/das Träger der Biobanken bzw. Archive*] übertragen. Ihre Proben werden nicht verkauft, der Träger kann aber für die Bereitstellung qualitätskontrollierter Biomaterialien von den jeweiligen Nutzern eine angemessene Aufwandsentschädigung verlangen. Ihr Recht, über die Verarbeitung Ihrer personenbezogenen Daten selbst zu bestimmen, bleibt von der Eigentumsübertragung unberührt. Trotz Eigentumsübertragung können Sie Ihre Einwilligung in die Datenverarbeitung jederzeit widerrufen (siehe Punkt 6) und die Vernichtung Ihrer Biomaterialien verlangen.

***Ende Modul Biomaterialien*]**

4. Erfolgt eine erneute Kontaktaufnahme mit Ihnen?

Zur Gewinnung zusätzlicher Informationen [*falls zutreffend:* oder Biomaterialien] von Ihnen kann es sinnvoll werden, zu einem späteren Zeitpunkt erneut Kontakt mit Ihnen aufzunehmen. Zudem kann die erneute Kontaktaufnahme dazu genutzt werden, z.B.

4.1

um von Ihnen mit Ihrer Zustimmung **zusätzliche, für wissenschaftliche Fragen relevante Informationen zu erfragen**, Sie über neue Forschungsvorhaben/Studien zu informieren und/oder Ihre Einwilligung in die Verknüpfung Ihrer Patientendaten mit medizinischen Informationen aus anderen Datenbanken einzuholen, oder

4.2

um Sie über **medizinische Zusatzbefunde zu informieren** (siehe oben Punkt 1.5).

Sie können die in 4.1 und 4.2 genannten Kontaktaufnahmen in der Einwilligungserklärung ablehnen („Recht auf Nichtwissen“).

4.3

Unabhängig davon kann eine Kontaktaufnahme erfolgen, um **Ihnen über Ihren behandelnden Arzt oder Ihren Hausarzt eine Rückmeldung** über Analyseergebnisse zu geben, die für Sie persönlich von erheblicher Bedeutung sein könnten (siehe oben Punkt 1.5).

5. Wie lange gilt Ihre Einwilligung?

Ihre Einwilligung in die Erhebung von Patientendaten [*falls zutreffend:* und zur Gewinnung von Biomaterialien] gilt – wenn Sie sie nicht vorher widerrufen (siehe weiter unten) – für einen Zeitraum von **fünf Jahren** ab Ihrer Einwilligungserklärung. Das bedeutet, dass in diesem Zeitraum [*in der/im Name der behandelnden Einrichtung*] mit vorheriger Ankündigung von Ihnen nochmals Daten [*falls zutreffend:* und Biomaterialien] gewonnen werden dürfen, ohne dass Sie erneut eine Einwilligungserklärung unterzeichnen müssten. Sollten Sie nach Ablauf von fünf Jahren wieder [*in der/im Name der behandelnden Einrichtung*] vorstellig werden, werden wir Sie erneut um Ihre Einwilligung bitten.

Ihre Einwilligung in die Verarbeitung und Nutzung der bisher erhobenen Daten [*falls zutreffend:* und gewonnenen Biomaterialien] bleibt über diesen Zeitraum hinaus wirksam (siehe Punkt 1.1).

6. Was beinhaltet Ihr Widerrufsrecht?

Ihre Einwilligung ist freiwillig!

Sie können Ihre Einwilligung zur weiteren Erhebung sowie zur wissenschaftlichen Nutzung Ihrer Patientendaten [**falls zutreffend:*** *sowie der von Ihnen zur Verfügung gestellten Biomaterialien] jederzeit ohne Angabe von Gründen und ohne nachteilige Folgen für Sie vollständig oder in Teilen widerrufen.***

Ein Widerruf bezieht sich dabei immer nur auf die künftige Verwendung Ihrer Patientendaten [*falls zutreffend:* und Biomaterialien]. Daten aus bereits durchgeführten Analysen können nachträglich nicht mehr entfernt werden.

Im Falle eines Widerrufs werden [*falls zutreffend:* die von Ihnen für die Forschung zur Verfügung gestellten Biomaterialien vernichtet und] Ihre auf Grundlage dieser Einwilligung gespeicherten Patientendaten gelöscht oder anonymisiert, sofern dies gesetzlich zulässig ist. Wenn eine Löschung nicht oder nicht mit zumutbarem technischem Aufwand möglich ist, werden Ihre Patientendaten anonymisiert, indem der Ihnen zugeordnete Identifizierungscode gelöscht wird. Die Anonymisierung Ihrer Patientendaten kann allerdings eine spätere Zuordnung von – insbesondere genetischen – Informationen zu Ihrer Person über andere Quellen niemals völlig ausschließen.

Sie können auch einzelne Teile der Einwilligungserklärung widerrufen, beispielsweise wenn Sie zwar die Patientendaten weiter der Forschung zur Verfügung stellen möchten, aber kein Interesse an einer weiteren Kontaktierung zwecks Nacherhebungen oder Studienteilnahmen haben.

Für einen Widerruf wenden Sie sich bitte an:

[*Adresse/Tel./Fax/Mail: Widerruf entgegennehmende Stelle/Einrichtung*]

7. Weitere Informationen und Rechte

Rechtsgrundlage für die Datenverarbeitung ist Ihre Einwilligung (Artikel 9 Absatz 2 a und Artikel 6 Absatz 1 a der Europäischen Datenschutz-Grundverordnung).

Für die Verarbeitung ihrer Patientendaten verantwortlich ist [*Name(n) der verantwortlichen Einrichtung und Kontaktdaten einfügen*].

Der zuständige Datenschutzbeauftragte der verantwortlichen Einrichtung ist erreichbar unter [*Kontaktdaten angeben*]

Sie haben die Möglichkeit, sich mit einer Beschwerde an jede Datenschutzaufsichtsbehörde zu wenden. Die zuständige Aufsichtsbehörde für Ihre behandelnde Einrichtung ist [*Name der zuständigen Datenschutzaufsichtsbehörde*].

Zudem haben Sie das Recht, Auskunft über die Sie betreffenden Patientendaten zu erhalten (auf Wunsch einschließlich einer unentgeltlichen Überlassung einer Kopie) sowie ggf. deren Berichtigung oder Löschung oder Einschränkung der Verarbeitung zu verlangen.

Sie haben weiter das Recht, von Ihnen bereitgestellte Daten in einem standardisierten elektronischen Format zu erhalten oder an eine von Ihnen genannte Stelle übermittelt zu bekommen (Recht auf Datenübertragbarkeit).

Einwilligungserklärung – Patientin/Patient

Einwilligung in die Nutzung von Patientendaten, [*falls zutreffend:* Krankenkassendaten und Biomaterialien (Gewebe und Körperflüssigkeiten)] für medizinische Forschungszwecke

1. Erhebung, Verarbeitung und wissenschaftliche Nutzung meiner Patientendaten, wie in der Patienteninformation beschrieben; dies umfasst

1.1

die Verarbeitung und Nutzung meiner Patientendaten für die medizinische Forschung ausschließlich wie in der Patienteninformation beschrieben und mit getrennter Verwaltung des Namens und anderer direkt identifizierender Daten (Codierung). Unter der Adresse www.medizininformatik-initiative.de/datennutzung kann ich mich für einen E-Mail-Verteiler registrieren, der per E-Mail über alle neuen Studien, die mit den Patientendaten durchgeführt werden, vor deren Durchführung informiert (siehe Punkte 1.1, 0 und 1.3 der Patienteninformation).

1.2

die wissenschaftliche Analyse und Nutzung meiner codierten Patientendaten durch Dritte wie z.B. durch andere Universitäten/Institute/forschende Unternehmen; dies kann auch eine Weitergabe für Forschungsprojekte im Ausland umfassen, wenn in diesen europäisches Datenschutzrecht gilt oder die Europäische Kommission ein angemessenes Datenschutzniveau bestätigt hat. An einem etwaigen kommerziellen Nutzen aus der Forschung werde ich nicht beteiligt. Vor einer Weitergabe an Forscher außerhalb meiner behandelnden Einrichtung erfolgt zudem eine weitere Ersetzung des internen Kennzeichens durch eine neue Zeichenkombination.

1.3

die Möglichkeit einer Zusammenführung meiner Patientendaten mit Daten in Datenbanken anderer Forschungspartner. **Voraussetzung ist, dass ich dieser Nutzung bei den entsprechenden Forschungspartnern auch zugestimmt habe.**

Ich willige ein in die Erhebung, Verarbeitung, Speicherung und wissenschaftliche Nutzung meiner **Patientendaten** wie in Punkt 1.1 bis 1.3 der Einwilligungserklärung und Punkt 1 der Patienteninformation beschrieben.

☐ Ja ☐ Nein

[*Falls zutreffend:*

Ich willige ein in die Verarbeitung und wissenschaftliche Nutzung meiner **Patientendaten, die im Rahmen früherer Behandlungen** erhoben wurden, wie in Punkt 1.1 bis 1.3 der Einwilligungserklärung und Punkt 1 der Patienteninformation beschrieben.

☐ Ja ☐ Nein

***Ende Modul Retrospektive Daten – Abschnitt „Patientendaten“*]**

[*Falls zutreffend:*

Meine Einwilligung umfasst auch die Übermittlung meiner **Patientendaten** in Länder, bei denen von der Europäischen Kommission kein angemessenes Datenschutzniveau festgestellt wurde. **Über die möglichen Risiken einer solchen Übermittlung bin ich aufgeklärt worden** (Punkt 1.3 in der Patienteninformation).

☐ Ja ☐ Nein

Ende Drittstaatenmodul – Abschnitt „Patientendaten“]

[*Falls zutreffend:*

2. Übertragung und wissenschaftliche Nutzung meiner Krankenkassendaten

Hiermit ermächtige ich meine Krankenkasse auf Anforderung durch [*zuständige Stelle*] Daten über von mir in Anspruch genommene ärztliche Leistungen in der ambulanten Versorgung und bei stationären Aufenthalten, über verordnete Heil- und Hilfsmittel sowie Arzneimittel und Angaben zum Bereich Pflege an [*Name der behandelnden Einrichtung*] so wie in der Patienteninformation beschrieben, zu übermitteln, und zwar:

2.1

Einmalig **rückwirkend für die Daten der vergangenen 5 Kalenderjahre**. Mit der dafür nötigen Übermittlung meiner Krankenversicherungs-Nummer an [*zuständige Stelle*] bin ich einverstanden

☐ Ja ☐ Nein

2.2

Für Daten **ab dem Datum meiner Unterschrift über einen Zeitraum von 5 Jahren**. Mit der dafür nötigen Übermittlung meiner Krankenversicherungs-Nr. an [*zuständige Stelle*] bin ich einverstanden

☐ Ja ☐ Nein

Ende Modul Kassendaten]

[***Falls zutreffend:***

3. Gewinnung, Lagerung und wissenschaftliche Nutzung meiner Biomaterialien (Gewebe und Körperflüssigkeiten), wie in der Patienteninformation beschrieben; dies umfasst

3.1

die Lagerung und Verarbeitung meiner Biomaterialien in [*der/dem Träger der Biobank(en) bzw. Archive*] für medizinische Forschungszwecke ausschließlich wie in der Patienteninformation beschrieben und mit getrennter Verwaltung des Namens und anderer direkt identifizierender Daten (Codierung, siehe Punkte 3.1 bis 3.3).

3.2

die wissenschaftliche Analyse meiner codierten Biomaterialien sowie deren Weitergabe und Nutzung durch Dritte z.B. Universitäten/Institute/forschende Unternehmen für genauer bestimmte und beantragte medizinische Forschungszwecke; dies kann auch eine Weitergabe für Forschungsprojekte im Ausland umfassen, wenn in diesen europäisches Datenschutzrecht gilt oder die Europäische Kommission ein angemessenes Datenschutzniveau bestätigt hat. Vor einer Weitergabe an Forscher außerhalb meiner behandelnden Einrichtung erfolgt zudem eine weitere Ersetzung des internen Kennzeichens durch eine neue Zeichenkombination.

Ebenso willige ich in die Möglichkeit einer Zusammenführung von Analyse-Daten meiner Biomaterialien mit Analyse-Daten in Datenbanken anderer Forschungspartner ein. **Voraussetzung ist, dass ich dieser Nutzung bei den entsprechenden Forschungspartnern auch zugestimmt habe**.

3.3

Das Eigentum an meinen Biomaterialien übertrage ich an [***den/die/das Träger der Biobank(en) bzw. Archive***]. Mein Recht, über die Verarbeitung meiner dem Biomaterial zu entnehmenden personenbezogenen Daten selbst zu bestimmen, bleibt von der Eigentumsübertragung unberührt (siehe Punkt 3.3 der Patienteninformation).

Ich willige ein in die Gewinnung, Lagerung und wissenschaftliche Nutzung meiner **Biomaterialien** (Gewebe und Körperflüssigkeiten), wie in Punkt 3.1 bis 3.3 der Einwilligungserklärung und Punkt 3 der Patienteninformation beschrieben.

☐ Ja ☐ Nein

[*Falls zutreffend:* Meine Einwilligung umfasst auch die Entnahme geringer zusätzlicher Mengen von Biomaterial bei einer sowieso stattfindenden Routine-Blutentnahme oder -Punktion in den unter Punkt 3.20 der Patienteninformation beschriebenen Grenzen.

☐ Ja ☐ Nein]

[***Falls zutreffend:***

Ich willige ein in die Verarbeitung und wissenschaftliche Nutzung meiner **Biomaterialien, die im Rahmen früherer Behandlungen** gewonnen wurden, wie in Punkt 3.1 bis 3.3 der Einwilligungserklärung und Punkt 3 der Patienteninformation beschrieben.

☐ Ja ☐ Nein

Ende Modul Retrospektive Daten – Abschnitt „Biomaterialien“]

[***Falls zutreffend:***

Meine Einwilligung umfasst auch die Weitergabe meiner **Biomaterialien** in Länder, bei denen von der Europäischen Kommission kein angemessenes Datenschutzniveau festgestellt wurde. **Über die möglichen Risiken einer solchen Weitergabe bin ich aufgeklärt worden** (Punkt 1.3 in der Patienteninformation).

☐ Ja ☐ Nein

Ende Drittstaatenmodul – Abschnitt „Biomaterialien“]

Ende Modul Biomaterialien]

4. Möglichkeit einer erneuten Kontaktaufnahme

4.1

Ich willige ein, dass ich von [*der/dem Name der behandelnden Einrichtung*] erneut kontaktiert werden darf, um gegebenenfalls zusätzliche für wissenschaftliche Fragen relevante Informationen [*falls zutreffend:* oder Biomaterialien] zur Verfügung zu stellen, um über neue Forschungsvorhaben/Studien informiert zu werden, und/oder um meine Einwilligung in die Verknüpfung meiner Patientendaten mit medizinischen Informationen aus anderen Datenbanken einzuholen (siehe Punkt 4.1 der Patienteninformation).

☐ Ja ☐ Nein

4.2

Ich willige ein, dass ich von [*der/dem Name der behandelnden Einrichtung*] wieder kontaktiert werden darf, um über medizinische Zusatzbefunde informiert zu werden (siehe Punkt 4.2 der Patienteninformation).

☐ Ja ☐ Nein

5. Geltungsdauer meiner Einwilligung

Meine Einwilligung in die Erhebung von Patientendaten [*falls zutreffend:* und in die Gewinnung von Biomaterialien] bei Aufenthalten [*in der/im Name der behandelnden Einrichtung*] gilt für einen **Zeitraum von fünf Jahren** ab meiner Einwilligungserklärung. Sollte ich nach Ablauf von fünf Jahren wieder in

[*der/dem Name der behandelnden Einrichtung*] vorstellig werden, kann ich erneut meine Einwilligung erteilen. Die Nutzung der von mir erhobenen Daten [*falls zutreffend:* und gewonnenen Biomaterialien] bleibt über diesen Zeitraum hinaus zulässig (Punkt 5 der Patienteninformation).

6. Widerrufsrecht

Meine Einwilligung ist **freiwillig!**

Ich kann meine Einwilligung jederzeit ohne Angabe von Gründen bei [*der/dem Name der behandelnden Einrichtung*] vollständig oder in Teilen widerrufen, ohne dass mir irgendwelche Nachteile entstehen.

Beim Widerruf werden [*falls zutreffend:* die für die Forschung verbliebenen Biomaterialien und] die auf Grundlage dieser Einwilligung gespeicherten Daten [*falls zutreffend:* vernichtet bzw.] gelöscht oder anonymisiert, sofern dies gesetzlich zulässig ist. Daten aus bereits durchgeführten Analysen können nicht mehr entfernt werden (Punkt 6 der Patienteninformation).

Ich wurde über die Nutzung meiner Patientendaten [**falls zutreffend:*****, Krankenkassendaten und Biomaterialien] sowie die damit verbundenen Risiken informiert und erteile im vorgenannten Rahmen meine Einwilligung. Ich hatte ausreichend Bedenkzeit und alle meine Fragen wurden zufriedenstellend beantwortet.***

Ich wurde darüber informiert, dass ich ein Exemplar der Patienteninformation und eine Kopie der unterschriebenen Einwilligungserklärung erhalten werde.

____
Ort, Datum

Vor- und Nachname Patient/in (Druckbuchstaben)	Unterschrift Patient/in

Ich habe das Aufklärungsgespräch geführt.

Vor- und Nachname Mitarbeiter/in (Druckbuchstaben)	Unterschrift Mitarbeiter/in
