# Supplementary material for: Broad consent in the emergency department: a cross sectional study
Source: Arch Public Health. 2025 Feb 18;83:44. doi: 10.1186/s13690-025-01529-z (PMC11834566; doi:10.1186/s13690-025-01529-z)
Supplement: Supplementary file 2 — Supplementary Material 2 [file 13690_2025_1529_MOESM2_ESM.pdf]

Record ID of the consented patients:

Entry is made by study assistant

Questionnaire for the patients
As part of the participation in "Broad Consent in the Emergency Department (BC-ED)

Firstly, we would like to as you questions about your “broad consent”, its understanding and its voluntary nature:

1. Did you understand everything in the patient information?

☐ Yes ☐ No

2. If ‘no’, what was the reason? (multiple answers possible)

- ☐ Too much information.
- ☐ Too long.
- ☐ I read too quickly.
- ☐ I'm not particularly interested in the content.
- ☐ The essentials were not clearly presented.
- ☐ I did not understand technical terms.
- ☐ Too little explanation.
- ☐ The language was too difficult.
- ☐ I was too excited.
- ☐ I didn't have enough time.
- ☐ Other: ____

3a. How do you rate the educational talk on the use of your data? (multiple answers possible)

- ☐ I would have liked a more detailed educational talk.
- ☐ The educational talk could have been shorter.
- ☐ I was given all the necessary information during the educational talk.
- ☐ All my questions were answered during the educational talk.
- ☐ I would have preferred to have been informed by a doctor.
- ☐ Unfortunately, I didn't have enough time to think about it.
- ☐ I had time to think about it and was able to ask all my questions.
- ☐ My questions were answered satisfactorily.
- ☐ I signed everything to finish quickly.
- ☐ Other: ____

3b. Do you currently feel sufficiently informed about the scientific use of patient data?

☐ Yes ☐ No

4a. How would you rate the educational talk on the use of your biomaterials? (multiple answers possible)

- ☐ I would have liked a more detailed educational talk.
- ☐ The educational talk could have been shorter.
- ☐ I was given all the necessary information during the educational talk.
- ☐ All my questions were answered during the educational talk.
- ☐ I would have preferred to have been informed by a doctor.
- ☐ Unfortunately, I didn't have enough time to think about it.
- ☐ I had time to think about it and was able to ask all my questions.
- ☐ My questions were answered satisfactorily.
- ☐ I signed everything to finish quickly.
- ☐ Other: ____

4b. Do you currently feel sufficiently informed about the scientific use of biomaterials?

☐ Yes ☐ No

5. What is the reason or motivation for your consent? (multiple answers possible)

☐ General support for research.

☐ Help for future patients.

☐ Own advantages through research.

☐ Feeling of connection with future patients.

☐ Gratitude towards the staff providing care.

☐ Hope for own advantages.

☐ On the recommendation of other people.

☐ Fear of poorer treatment if consent is not given.

☐ No specific reasons.

☐ Other: ____

6. Have you already participated in a study in the field of medical research?

☐ Yes ☐ No

7. Has anyone you know well or a member of your family ever taken part in a medical research study?

☐ Yes ☐ No

8. Do you work in the healthcare sector?

☐ Yes ☐ No

8a. If 'yes', in which area?

☐ Medical doctor.

☐ Healthcare professionals (e.g. nurses, therapists, paramedics).

☐ Staff not directly involved in patient care (e.g. administrative staff, medical technicians, scientists, IT staff).

9. Did you have sufficient time to consider the decision to give consent?

☐ Yes ☐ No

10a. Did you understand the content of the consent before you decided to give your consent?

☐ Not at all.

☐ Rather not.

☐ Yes, mostly.

☐ Yes, completely.

10b. Did you feel that the benefits of taking part in this study were explained to you?

☐ Not at all.

☐ Rather not.

☐ Yes, mostly.

☐ Yes, completely.

10c. Did you feel that the inconveniences and risks of taking part in this study were explained to you?

☐ Not at all.

☐ Rather not.

☐ Yes, mostly.

☐ Yes, completely.

10d. Did you feel that the trial staff were available and willing to answer any questions or concerns you had about the trial?

☐ Not at all.

☐ Rather not.

☐ Yes, mostly.

☐ Yes, completely.

10e. Were your questions and concerns answered satisfactorily by the study staff?

☐ Not at all

☐ Rather not.

☐ Yes, mostly.

☐ Yes, completely.

11. Were your questions and concerns answered satisfactorily by the study staff?

11aI. Patient information

☐ Yes, completely.

☐ Partially.

☐ Not at all.

☐ Not clear.

11aII. The scope of the written patient information is...

☐ Not detailed enough.

☐ Just right.

☐ Too detailed.

☐ I haven't read it.

11aIII. Is the content of the written patient information written in an understandable way?

☐ Overall yes.

☐ Mostly yes.

☐ Mostly no.

☐ Overall no.

☐ I have not read them.

11bI. Information video

☐ Yes, completely.

☐ Partially.

☐ Not at all.

☐ Not clear.

11bII. The scope of the information video is......

☐ Not detailed enough.

☐ Just right.

☐ Too detailed.

☐ I haven't looked at it.

11bIII. Is the content of the information video presented clearly?

☐ Overall yes.

☐ Mostly yes.

☐ Mostly no.

☐ Overall no.

☐ I didn’t watch.

11c. Other sources of information: ______________________________________

12. Please indicate which modules of the 'Broad Consent' (BC) you have just consented to.

12a. Patient data

☐ Current

☐ Data already available, 5 years retrospectively

12b. Health insurance data

☐ Data already available, 5 years retrospectively

☐ Data collected in the future, up to a further 5 years

12c. Biomaterial

☐ Current

☐ Samples already taken, 5 years retrospectively

12d. Recontacting

☐ For further questions

☐ Information about additional medical findings

12e. I have not agreed to any of the modules because... (multiple answers possible):

☐ I have not had enough time to think about it.

☐ I am generally not interested in donating data.

☐ I have concerns about privacy.

☐ I did not want to make this decision in the stressful medical situation of my emergency department stay.

☐ Other: ____

13. If you have consented to share data relating to your current emergency department treatment, which data will this specifically include?

☐ Data on my treatment in the emergency department **without** data from the possible subsequent inpatient stay.

☐ Data on my treatment in the emergency department **and** data from the possible subsequent inpatient stay.

☐ I do not know.

14. Please use the space below to formulate your further comments on the BC or the BC-information process:

____

____

Part two follows on the next page.

In the second part, we would like to collect a few more details about you:

S1. Age: _____________

S2. Gender:

- ☐ Female
- ☐ Male
- ☐ Other (Please specify your gender yourself):

  ____

- ☐ I prefer not to answer

S3. What gender were you assigned at birth?

- ☐ Female
- ☐ Male
- ☐ Other
- ☐ I prefer not to answer

S4. Country of birth

- ☐ Germany
- ☐ EU country

  If ‘yes’, which one: ___________________________

- ☐ Non-EU country

  If ‘yes’, which one: ___________________________

S5a. Which language do you prefer?

____

- ☐ I prefer not to answer

S5b. How well do you consider yourself to speak German?

- ☐ Mother language
- ☐ Fluent
- ☐ Good knowledge
- ☐ A little
- ☐ I prefer not to answer

S6. What is your highest school-leaving qualification?

☐ A-levels, general higher education entrance qualification

☐ Advanced technical college entrance qualification, specialised secondary school entrance qualification

☐ Intermediate school leaving certificate

☐ Secondary/elementary school

☐ Finished school without leaving certificate

☐ Other qualification (e.g. obtained abroad)

☐ I prefer not to answer

S7. What is your highest educational qualification?

☐ University

☐ University of applied sciences, engineering school

☐ Technical school (vocational or technical

☐ Apprenticeship (vocational training)

☐ No qualification or still in vocational training

☐ Other educational qualification

☐ I prefer not to answer

S8. Are you currently...

☐ in full-time employment

☐ in part-time employment

☐ Self-employed

☐ Occasionally or irregularly employed

☐ On parental leave / maternity leave

☐ Pupil and student

☐ Not employed

☐ Retired, pensioner, early retiree

☐ I prefer not to answer

S9. How many people live in your household, including you?

☐ Total number of people living in the household: _______

☐ I prefer not to answer

S10. How many people in your household are under the age of 14?

____

S11. Are you the main earner in your household?

- ☐ Yes
- ☐ No
- ☐ I don’t have an answer for that
- ☐ I prefer not to answer

S12. What is the total monthly net income of your household?

- ☐ Above 2500 Euro
- ☐ About 2500 Euro
- ☐ Below 2500 Euro
- ☐ I prefer not to answer

S13. Do you have a nursing care level?

1	2	3	4	5	No
☐	☐	☐	☐	☐	☐

- ☐ I prefer not to answer

S14. What is your current marital status?

- ☐ Single
- ☐ Divorced
- ☐ Married (living together)
- ☐ Married (living apart)
- ☐ Registered partnership (living together)
- ☐ Registered partnership (living apart)
- ☐ Widowed
- ☐ I prefer not to answer

S15. How do you identify yourself?

☐ Queer

☐ Lesbian

☐ Gay

☐ Bisexual

☐ Heterosexual

☐ Other sexuality

☐ I prefer not to answer

S16. Where do you currently live?

☐ In an apartment or house (owned, rented or with relatives)

☐ Assisted living (e.g. retirement apartments, retirement homes, senior residences, senior-friendly living)

☐ Inpatient care

☐ Refugee accommodation

☐ No permanent residence

☐ I prefer not to answer

☐ Other: ____

Thank you very much for your participation in this survey.

If you have any questions or queries, please do not hesitate to contact the study team, who will be happy to assist you.

Record ID der eingewilligten Patient*innen:

Eintrag erfolgt durch Studienassistenz

Fragebogen für die Patient*innen
Im Rahmen der Teilnahme an „Die Breite Einwilligung in der Notaufnahme (BC-ED)

Zuerst möchten wir Ihnen Fragen zu Ihrer „Breiten Einwilligung“, deren Verständnis und Freiwilligkeit stellen:

1. Haben Sie in der Patient*innen-Information alles verstanden?

☐ Ja ☐ Nein

2. Wenn „nein“, woran lag das? (Mehrfachnennung möglich)

☐ Zu viele Informationen.

☐ Zu lang.

☐ Ich habe zu schnell gelesen.

☐ Mich interessiert der Inhalt nicht besonders.

☐ Das Wesentliche wurde nicht klar dargestellt.

☐ Fachausdrücke nicht verstanden.

☐ Zu wenig Erklärung.

☐ Zu schwierige Sprache.

☐ Ich war zu aufgeregt.

☐ Ich hatte nicht genug Zeit.

☐ Sonstiges: ____

3a. Wie schätzen Sie das Aufklärungsgespräch zur Nutzung Ihrer Daten ein? (Mehrfachnennungen möglich)

☐ Ich hätte mir ein ausführlicheres Gespräch gewünscht.

☐ Das Gespräch hätte kürzer sein können.

☐ Im Gespräch wurden mir alle notwendigen Informationen vermittelt.

☐ Alle meine Fragen wurden im Gespräch beantwortet.

☐ Ich wäre lieber von einem Arzt/einer Ärztin aufgeklärt worden.

☐ Ich hatte leider nicht genug Zeit mir Gedanken zu machen.

☐ Ich hatte Zeit zum Überlegen und konnte alle meine Fragen stellen.

☐ Meine Fragen wurden zufriedenstellend beantwortet.

☐ Ich habe alles unterschrieben, um schnell fertig zu werden.

☐ Sonstiges: ____

3b. Fühlen Sie sich zum jetzigen Zeitpunkt ausreichend über die wissenschaftliche Nutzung von Patient*innendaten informiert?

☐ Ja ☐ Nein

4a. Wie schätzen Sie das Aufklärungsgespräch zur Nutzung Ihrer Biomaterialien ein?

(Mehrfachnennungen möglich)

☐ Ich hätte mir ein ausführlicheres Gespräch gewünscht.

☐ Das Gespräch hätte kürzer sein können.

☐ Im Gespräch wurden mir alle notwendigen Informationen vermittelt.

☐ Alle meine Fragen wurden im Gespräch beantwortet.

☐ Ich wäre lieber von einem Arzt/einer Ärztin aufgeklärt worden.

☐ Ich hatte leider nicht genug Zeit mir Gedanken zu machen.

☐ Ich hatte Zeit zum Überlegen und konnte alle meine Fragen stellen.

☐ Meine Fragen wurden zufriedenstellend beantwortet.

☐ Ich habe alles unterschrieben, um schnell fertig zu werden.

☐ Sonstiges: ____

4b. Fühlen Sie sich zum jetzigen Zeitpunkt ausreichend über die wissenschaftliche Nutzung von Biomaterialien informiert?

☐ Ja ☐ Nein

5. Was ist der Grund oder die Motivation für Ihre Einwilligung? (Mehrfachnennung möglich)

☐ generelle Unterstützung der Forschung

☐ Hilfe für zukünftige Patient*innen

☐ eigene Vorteile durch Forschung

☐ Gefühl von Verbundenheit mit zukünftigen Patient*innen

☐ Dankbarkeit gegenüber dem versorgenden Personal

☐ Hoffen auf eigene Vorteile

☐ Auf Empfehlung anderer Personen

☐ Befürchtung einer schlechteren Behandlung bei Nicht-Einwilligung

☐ keine spezifischen Gründe

☐ Andere: ____

6. Haben Sie bereits an einer Studie im Bereich der medizinischen Forschung teilgenommen?

☐ Ja ☐ Nein

7. Hat jemand, den Sie gut kennen oder ein Mitglied Ihrer Familie bereits an einer Studie im Bereich der medizinischen Forschung teilgenommen?

☐ Ja ☐ Nein

8. Sind Sie im Gesundheitswesen tätig?

☐ Ja ☐ Nein

8a. Wenn „ja", in welchem Bereich?

☐ ärztlich

☐ Angehörige/r eines Pflegefachberufs (z. B. Pflegepersonal, Therapeut, Rettungskraft)

☐ Personal außerhalb der direkten Patientenversorgung (z. B. administrativer Mitarbeiter, Medizintechniker, Wissenschaftler, IT-Mitarbeiter)

9. Stand Ihnen ausreichend Bedenkzeit für die Entscheidung zur Einwilligung zur Verfügung?

☐ Ja ☐ Nein

10a. Haben Sie die Inhalte der Einwilligung verstanden, bevor Sie sich zur Zustimmung entschlossen haben?

☐ Überhaupt nicht

☐ eher nicht

☐ ja, überwiegend

☐ ja, vollständig

10b. Haben Sie den Eindruck, dass die Vorteile der Teilnahme an dieser Studie erklärt wurden?

☐ Überhaupt nicht

☐ eher nicht

☐ ja, überwiegend

☐ ja, vollständig

10c. Haben Sie den Eindruck, dass Ihnen die Unannehmlichkeiten und Risiken der Teilnahme an dieser Studie erklärt wurden?

☐ Überhaupt nicht

☐ eher nicht

☐ ja, überwiegend

☐ ja, vollständig

10d. Hatten Sie das Gefühl, dass das Studienpersonal verfügbar und bereit war, Ihre Fragen oder Bedenken bezüglich der Studie zu beantworten?

☐ Überhaupt nicht

☐ eher nicht

☐ ja, überwiegend

☐ ja, vollständig

10e. Wurden auf Ihre Fragen und Bedenken durch das Studienpersonal zufriedenstellend eingegangen?

☐ Überhaupt nicht

☐ eher nicht

☐ ja, überwiegend

☐ ja, vollständig

11. Über welche Materialien haben Sie sich über die „Breite Einwilligung“ informieren können?

11aI. Patient*innen-information

☐ ja, komplett

☐ teilweise

☐ gar nicht

☐ nicht klar

11aII. Der Umfang der schriftlichen Patient*innen-Information ist…

☐ Nicht ausführlich genug.

☐ Genau richtig.

☐ Zu ausführlich.

☐ Ich habe sie nicht gelesen.

11aIII. Ist der Inhalt der schriftlichen Patient*innen-Information verständlich geschrieben?

☐ Insgesamt ja

☐ überwiegend ja

☐ überwiegend nein

☐ insgesamt nein

☐ Ich habe sie nicht gelesen.

11bI. Informationsvideo

☐ ja, komplett

☐ teilweise

☐ gar nicht

☐ nicht klar

11bII. Der Umfang des Informationsvideos ist…

☐ Nicht ausführlich genug.

☐ Genau richtig.

☐ Zu ausführlich.

☐ Ich habe es nicht angesehen.

11bIII. Ist der Inhalt des Informationsvideos verständlich dargestellt?

☐ Insgesamt ja

☐ überwiegend ja

☐ überwiegend nein

☐ insgesamt nein

☐ Ich habe es nicht angesehen.

11c. Andere Informationsquellen: ____________________________________

12. Bitte geben Sie wieder, in welche Module der „Breiten Einwilligung“ (Broad Consent – BC) Sie soeben eingewilligt haben.

12a. Patientendaten

- ☐ Aktuell
- ☐ bereits vorliegende Daten, 5 Jahre rückwirkend

12b. Krankenkassen-/Versicherungsdaten

- ☐ bereits vorliegende Daten, 5 Jahre rückwirkend
- ☐ zukünftig erhobene Daten, bis zu weiteren 5 Jahren

12c. Biomaterial

- ☐ Aktuell
- ☐ bereits entnommene Proben, 5 Jahre rückwirkend

12d. Rekontaktierung

- ☐ bei weiteren Rückfragen
- ☐ Information über medizinische Zusatzbefunde

12e. ich habe in keines der Module eingewilligt, weil… (Mehrfachnennung möglich):

- ☐ ich zu wenig Bedenkzeit hatte.
- ☐ ich generell kein Interesse an einer Datenspende habe.
- ☐ ich bezüglich Datenschutz Bedenken habe.
- ☐ ich in der belastenden medizinischen Situation meines Notaufnahmeaufenthaltes diese Entscheidung nicht fällen wollte.
- ☐ Anderes: ____

13. Falls Sie der Datenspende Ihrer aktuellen Behandlung in der Notaufnahme zugestimmt haben, welche betrifft dies konkret?

- ☐ Daten zur meiner Behandlung in der Notaufnahme **ohne** Daten aus dem möglicherweise folgenden stationären Aufenthalt.
- ☐ Daten zur meiner Behandlung in der Notaufnahme **und** Daten aus dem möglicherweise folgenden stationären Aufenthalt.
- ☐ Ich weiß nicht.

14. Bitte nutzen Sie den nachstehenden Platz, um Ihre weiteren Anmerkungen zum BC oder dem Aufklärungsprozess zu formulieren:

____

____

Auf der nächsten Seite folgt der zweite Teil.

Im zweiten Teil möchten wir noch ein paar Angaben zu Ihrer Person erheben:

S1. Alter: _____________

S2. Geschlecht:

☐ Weiblich

☐ Männlich

☐ Andere Angabe (Bitte spezifizieren Sie ihr Geschlecht selbst):

____

☐ Ich bevorzuge nicht zu antworten

S3. Welches Geschlecht wurde Ihnen bei Geburt zugewiesen?

☐ Weiblich

☐ Männlich

☐ Andere

☐ Ich bevorzuge nicht zu antworten

S4. Geburtsland

☐ Deutschland

☐ EU Land

Wenn „ja“, welches: ___________________________

☐ Nicht-EU-Land

Wenn „ja“, welches: ___________________________

S5a. Welche Sprache bevorzugen Sie?

____

☐ Ich bevorzuge nicht zu antworten

S5b. Wie gut sprechen Sie selbsteingeschätzt Deutsch?

☐ Muttersprache

☐ Fließend

☐ Gute Kenntnisse

☐ Ein wenig

☐ Ich bevorzuge nicht zu antworten

S6. Welchen höchsten Schulabschluss haben Sie?

- ☐ Abitur, Allgemeine Hochschulreife
- ☐ Fachhochschulreife, Fachoberschulreife
- ☐ Mittlere Reife
- ☐ Haupt-/Volksschule
- ☐ Schule beendet ohne Abschluss
- ☐ Anderer Schulabschluss (z. B. Im Ausland erworben)
- ☐ Ich bevorzuge nicht zu antworten

S7. Welchen höchsten Ausbildungsabschluss haben Sie?

- ☐ Universität
- ☐ Fachhochschule, Ingenieurschule
- ☐ Fachschule (Berufs- oder Fachakademie)
- ☐ Lehre (beruflich-betriebliche Ausbildung)
- ☐ Keinen Abschluss oder noch in beruflicher Ausbildung
- ☐ Anderen Ausbildungsabschluss
- ☐ Ich bevorzuge nicht zu antworten

S8. Sind Sie derzeit…

- ☐ Vollzeit erwerbstätig
- ☐ Teilzeit erwerbstätig
- ☐ selbständige Tätigkeit
- ☐ Gelegentlich oder unregelmäßig erwerbstätig
- ☐ In Elternzeit / Mutterschutz
- ☐ Schüler und Studierende
- ☐ Nicht erwerbstätig
- ☐ Berentet, Pension, Frühruhestand
- ☐ Ich bevorzuge nicht zu antworten

S9. Wie viele Personen leben in Ihrem Haushalt, Sie eingeschlossen?

- ☐ Anzahl der im Haushalt lebenden Personen insgesamt: _______
- ☐ Ich bevorzuge nicht zu antworten

S10. Wie viele Personen in Ihrem Haushalt sind jünger als 14 Jahre?

____

S11. Sind Sie Hauptverdiener*in in Ihrem Haushalt?

- ☐ Ja
- ☐ Nein
- ☐ Ich kann das nicht beantworten
- ☐ Ich bevorzuge nicht zu antworten

S12. Wie hoch ist in etwa das monatliche Netto-Einkommen Ihres Haushaltes insgesamt?

- ☐ Über 2500 Euro
- ☐ Bei etwa 2500 Euro
- ☐ Unter 2500 Euro
- ☐ Ich bevorzuge nicht zu antworten

S13. Besteht eine Pflegestufe?

1	2	3	4	5	Nein
☐	☐	☐	☐	☐	☐

- ☐ Ich bevorzuge nicht zu antworten

S14. Welchen Familienstand haben Sie aktuell?

- ☐ Ledig
- ☐ Geschieden
- ☐ Verheiratet (zusammenlebend)
- ☐ Verheiratet (getrennt lebend)
- ☐ Eingetragene Partnerschaft (zusammenlebend)
- ☐ Eingetragene Partnerschaft (getrennt lebend)
- ☐ Verwitwet
- ☐ Ich bevorzuge nicht zu antworten

S15. Wie identifizieren Sie sich?

☐ Queer

☐ Lesbisch

☐ Schwul

☐ Bisexuell

☐ Heterosexuell

☐ Andere Sexualität

☐ Ich bevorzuge nicht zu antworten

S16. Wo wohnen Sie zurzeit?

☐ In einer Wohnung oder im Haus (Eigentum, zur Miete oder bei Verwandten)

☐ Betreutes Wohnen (zB. Alters-WG, Altersheime, Seniorenresidenzen, seniorengerechtes Wohnen)

☐ stationäre Pflege

☐ Flüchtlingsunterkunft

☐ kein fester Wohnsitz

☐ Ich bevorzuge nicht zu antworten

☐ Sonstiges: ____

Wir danken Ihnen herzlich für Ihre Teilnahme an dieser Befragung.

Bei Fragen und Unklarheiten können Sie sich jederzeit an die Studienmitarbeiter*innen wenden, die Sie gerne unterstützen werden.
