# Supplementary material for: Broad consent in the emergency department: a cross sectional study
Source: Arch Public Health. 2025 Feb 18;83:44. doi: 10.1186/s13690-025-01529-z (PMC11834566; doi:10.1186/s13690-025-01529-z)
Supplement: Supplementary file 3 — Supplementary Material 3 [file 13690_2025_1529_MOESM3_ESM.pdf]

Sequential number of every fifth patient: ____________________

Record ID (REDCap®): ____________________

Date and time of administrative admission: Date (DD.MM.YYYY) __ __. ___ ___ . ___ ___ ___ ___ Time (hh:mm) __ __: __ __

Survey form for the information process for all patients approached as part of participation in Broad Consent in the Emergency Department (BC-ED)"

(study assistant fills in)

Questions 1-3 must be documented for every fifth patient (SCREENING)

1. Examination of the inclusion criteria:

1a. Age: __________

1b. Ability to give consent:

- ☐ Yes
- ☐ No
- ☐ Cannot be assessed

1bI. If 'no', why not able to give consent (multiple answers possible)

- ☐ Language barrier
- ☐ Sedated
- ☐ Dementia
- ☐ Traumatic brain injury
- ☐ Too much pain
- ☐ Intubated
- ☐ Being cared for
- ☐ Isolation
- ☐ Inability to understand the nature, importance and scope of the study
- ☐ Child
- ☐ Deceased
- ☐ Other: ______________________________

2. if eligible - was the patient included/addressed?

☐ Yes

☐ No

2a. If 'no', why not enrolled / could not be contacted?

☐ Patient was transferred directly / transferred to the operating theatre

☐ Patient had already left the emergency department before contacting the study assistant

☐ Patient was transferred to another clinic

☐ For medical reasons (too poor or deteriorating health; urgent medical care with no opportunity to talk afterwards)

☐ Patient died

☐ Other: ______________________________

3. if admission/approach has taken place - has the patient verbally agreed to informed consent to participate in BC-ED?

☐ Yes

☐ No

3a. If "no", why was consent not given

☐ Patient generally refuses to participate in the study

☐ Patient has no interest in the study

☐ Patient does not feel able to do so at the moment

☐ Other: ______________________________

4. Direct Consenting:

☐ Yes

☐ No

Delayed Consenting:

☐ Yes

☐ No

5a. Date and time of **start of clarification** in the BC:

Date (DD.MM.YYYY) __ __. __ __ . __ __ __ __ Time (hh:mm) __ __: __ __

5b. Date and time of the **end of the educational talk** in the BC:

Date (DD.MM.YYYY) __ __. __ __ . __ __ __ __ Time (hh:mm) __ __: __ __

5c. Date and time of **acceptance/rejection** of the BC:

Date (DD.MM.YYYY) __ __. __ __ . __ __ __ __ Time (hh:mm) __ __: __ __

5d. Interruptions?

☐ Yes – estimated duration (5 min interval) _____________________

☐ No

5e. Cancellation?

☐ Yes – for medical reasons (deterioration of health; urgent medical attention without the possibility of continuing the interview afterwards)

☐ Yes – at the patient's request (for personal reasons)

☐ Yes – the patient could not be found (due to transfer, leaving the NA)

---

6. Which modules of the BC have been consented to?

6a. Patient data

☐ Current

☐ 5 years retrospectively

6b. Health insurance data

☐ 5 years retrospectively

☐ up to a further 5 years

6c. Biomaterial

☐ Current

☐ 5 years retrospectively

6d. Recontacting

☐ For further questions

☐ Information about additional medical findings

6e. None of the BC modules have been consented to. ☐

7. Did the patient wish to have time for consideration?

☐ Yes

☐ No

If "yes", how long (estimated in minutes): _____________________

8. Where did the educational talk take place?

- ☐ Waiting area, seated
- ☐ Waiting area, lying down
- ☐ Separate room
- ☐ Treatment room
- ☐ Shock room
- ☐ Other location: ___________________________________

9. Which materials were used to inform the patient about the "broad consent"?

9a. Patient information

- ☐ Yes, completely.
- ☐ Partially.
- ☐ Not at all.
- ☐ Not clear.

9b. Video

- ☐ Yes, completely.
- ☐ Partially.
- ☐ Not at all.
- ☐ Not clear.

9c. Other sources of information: ____

10. What questions did the patient have about the content of the BC?

____

____

____

11. Do you have any further comments on the information process?

____

____

____

Fortlaufende Nummer jeder/s fünften Patient*in: ____________________

Record ID (REDCap®): ____________________

Datum und Uhrzeit der administrativen Aufnahme: Datum (TT.MM.JJJJ) __ __. __ __ . __ __ __ __ Uhrzeit (hh:mm) __ __: __ __

Erhebungsbogen für den Aufklärungsverlauf bei allen angesprochenen Patient*innen im Rahmen der Teilnahme an „Broad Consent/ Breiten Einwilligung“ in der Notaufnahme (BC-ED)“

(Studienassistenz füllt aus)

Fragen 1-3 sind für jede/n fünfte/n Patient*in zu dokumentieren (SCREENING)

1. Prüfung der <u>Einschlusskriterien:</u>

1a. Alter: __________

1b. Einwilligungsfähig:

- ☐ ja
- ☐ nein
- ☐ kann nicht beurteilt werden

1bI. Wenn „nein“, warum nicht einwilligungsfähig (Mehrfachnennung möglich)

- ☐ Sprachbarriere
- ☐ Sediert
- ☐ Dement
- ☐ Schädel-Hirn-Trauma
- ☐ Zu starke Schmerzen
- ☐ Intubiert
- ☐ Betreut
- ☐ Isolation
- ☐ Fehlende Fähigkeit für die Erfassung der Art, Bedeutung und Tragweite der Studie
- ☐ Kind
- ☐ Verstorben
- ☐ Anderes: ______________________________

2. wenn einschlussfähig - erfolgte der Einschluss/ die Ansprache der/des Patient*in?

- ☐ ja
- ☐ nein

2a. Wenn „nein“, warum nicht einschlussfähig/ keine Ansprache möglich

- ☐ Patient*in wurde direkt weiterverlegt / in den OP verlegt
- ☐ Patient*in hat die Notaufnahme bereits vor Kontakt mit der Studienassistenz verlassen
- ☐ Patient*in wurde in eine andere Klinik weiterverlegt
- ☐ aus medizinischen Gründen (zu schlechter oder Verschlechterung des Gesundheitszustandes; dringende medizinische Versorgung ohne Möglichkeit danach das Gespräch zu führen)
- ☐ Patient*in verstorben
- ☐ Anderes: ______________________________

3. wenn Einschluss/Ansprache erfolgt - hat die/der Patient*in der informierten Einwilligung zur Teilnahme am BC-ED mündlich zugestimmt?

- ☐ ja
- ☐ nein

3a. Wenn „nein“, warum nicht eingewilligt

- ☐ Patient*in lehnt eine Studienteilnahme prinzipiell ab
- ☐ Patient*in hat kein Interesse an dieser Studie
- ☐ Patient*in fühlt sich aktuell nicht in der Lage
- ☐ Anderes: ______________________________

4. Direktes Consenting:

☐ ja

☐ nein

Verzögertes Consenting:

☐ ja

☐ nein

5a. Datum und Uhrzeit **Beginn der Aufklärung** in den BC:

Datum (TT.MM.JJJJ) __ __. __ __ . __ __ __ __ Uhrzeit (hh:mm) __ __: __ __

5b. Datum und Uhrzeit **Ende Aufklärungsgespräch** in den BC:

Datum (TT.MM.JJJJ) __ __. __ __ . __ __ __ __ Uhrzeit (hh:mm) __ __: __ __

5c. Datum und Uhrzeit der **Einwilligung/ Ablehnung** in den BC:

Datum (TT.MM.JJJJ) __ __. __ __ . __ __ __ __ Uhrzeit (hh:mm) __ __: __ __

5d. Unterbrechungen?

☐ ja – geschätzte Dauer (5 min Intervall) ______________________

☐ nein

5e. Abbruch?

☐ ja – aus medizinischen Gründen (Verschlechterung des Gesundheitszustandes; dringende medizinische Versorgung ohne Möglichkeit danach das Gespräch weiter zu führen)

☐ ja – durch die/den Patient*in gewünscht (aus persönlichen Gründen)

☐ ja – die/der Patient*in war nicht mehr anzutreffen (durch Verlegung, Verlassen der NA)

---

6. In welche Module des BC wurde eingewilligt?

6a. Patientendaten

☐ Aktuell

☐ 5 Jahre rückwirkend

6b. Krankenkassen-/Versicherungsdaten

☐ 5 Jahre rückwirkend

☐ bis zu weiteren 5 Jahren

6c. Biomaterial

☐ Aktuell

☐ 5 Jahre rückwirkend

6d. Rekontaktierung

☐ weitere Fragen

☐ Information über medizinische Zusatzbefunde

6e. Es wurde in keines der Module des BC eingewilligt. ☐

7. Wünschte der/die Patient*in <u>Bedenkzeit</u>?

☐ ja

☐ nein

Wenn „ja", wie lange (geschätzt in min): ______________________

8. Wo fand die Aufklärung statt?

- ☐ Wartebereich sitzend
- ☐ Wartebereich liegend
- ☐ Separate Räumlichkeit
- ☐ Behandlungszimmer
- ☐ Schockraum
- ☐ Anderer Ort: ________________________________

9. Über welche <u>Materialien</u> wurde die/der Patient*in über die „Breite Einwilligung“ informiert?

9a. Patient*innen-information

- ☐ ja, komplett
- ☐ teilweise
- ☐ gar nicht
- ☐ nicht klar

9b. Video

- ☐ ja, komplett
- ☐ teilweise
- ☐ gar nicht
- ☐ nicht klar

9c. Andere Informationsquellen: ____________________________________

10. Welche <u>Rückfragen</u> zum Inhalt des BC hatte der/die Patient*in?

____

____

____

11. Haben Sie weitere <u>Anmerkungen</u> zum Aufklärungsprozess?

____

____

____
