# Supplementary material for: Broad consent in the emergency department: a cross sectional study
Source: Arch Public Health. 2025 Feb 18;83:44. doi: 10.1186/s13690-025-01529-z (PMC11834566; doi:10.1186/s13690-025-01529-z)
Supplement: Supplementary file 5 — Supplementary Material 5 [file 13690_2025_1529_MOESM5_ESM.docx]

**Supplement 5**

*Supplement Tab. 1: Answers of the patient questionnaire after consenting regarding understanding, recall, voluntariness, satisfaction with the consent procedure. Reported are absolute values and relative proportions.*

| Topic | Question | Answers | Absolute counts | Unless otherwise stated relative proportion based on n=225 |
| --- | --- | --- | --- | --- |
| Understanding | Did you understand the patient information? | Yes | 202 | 89.8 |
|  |  | No | 20 | 8.9 |
|  |  | Missing | 3 | 1.3 |
|  |  |  |  |  |
|  |  | No - too long | 12 | 5.3 |
|  |  | No - too much information | 10 | 4.4 |
|  |  | No - too difficult language | 8 | 3.6 |
|  |  | No - technical terms not understood | 3 | 1.3 |
|  |  | No - the essentials were not clearly presented | 2 | 0.9 |
|  |  | No - I was too nervous | 2 | 0.9 |
|  |  | No - I read too fast | 1 | 0.4 |
|  |  | No - I am not particularly interested in the content | 0 | 0.0 |
|  |  | No - too little explanation | 0 | 0.0 |
|  |  | No - I did not have enough time | 0 | 0.0 |
| Informative talk | How do you rate the informative talk about the use of your data? | I would have liked to have had a more detailed conversation. | 6 | 2.7 |
|  |  | The conversation could have been shorter. | 8 | 3.6 |
|  |  | During the interview I was given all the necessary information. | 161 | 71.6 |
|  |  | All my questions were answered in the conversation. | 93 | 41.3 |
|  |  | I would have preferred to be informed by a doctor. | 3 | 1.3 |
|  |  | Unfortunately. I did not have enough time to think about it. | 8 | 3.6 |
|  |  | I had time to think and could ask all my questions. | 69 | 30.7 |
|  |  | My questions were answered satisfactorily. | 79 | 35.1 |
|  |  | I signed everything to finish quickly. | 9 | 4.0 |
|  |  | Free text | 12 | 5.3 |
|  | Do you currently feel sufficiently informed about the scientific use of patient data? | Yes | 202 | 89.8 |
|  |  | No | 12 | 5.3 |
|  |  | Missing | 11 | 4.9 |
|  | How do you rate the informative talk about the use of your biomaterial? (n=168 as no Biomaterial module in one center was included) | I would have liked to have had a more detailed conversation. | 6 | 3.6 |
|  |  | The conversation could have been shorter. | 5 | 3.0 |
|  |  | During the interview I was given all the necessary information. | 103 | 61.3 |
|  |  | All my questions were answered in the conversation. | 52 | 31.0 |
|  |  | I would have preferred to be informed by a doctor. | 5 | 3.0 |
|  |  | Unfortunately, I did not have enough time to think about it. | 12 | 7.1 |
|  |  | I had time to think and could ask all my questions. | 32 | 19.0 |
|  |  | My questions were answered satisfactorily. | 50 | 29.8 |
|  |  | I signed everything to finish quickly. | 6 | 3.6 |
|  |  | Free text | 11 | 6.5 |
|  | Do you currently feel sufficiently informed about the scientific use of biomaterial? (n=168 as no Biomaterial module in one center was included) | Yes | 144 | 85.7 |
|  |  | No | 20 | 11.9 |
|  |  | Missing | 4 | 2.4 |
| Former participation in research, profession, reflection period | Have you already participated in a study in the field of medical research? | Yes | 61 | 27.1 |
|  |  | No | 162 | 72.0 |
|  |  | Missing | 2 | 0.9 |
|  | Has someone you know well or a member of your family already taken part in a study in the field of medical research? | Yes | 45 | 20.0 |
|  |  | No | 174 | 77.3 |
|  |  | Missing | 6 | 2.7 |
|  | Do you work in the health care sector? (n=25 valid; n=3missing) | Yes | 28 | 12.4 |
|  |  | If yes, in which area? - Medical doctor | 2 | 7.1* |
|  |  | If yes, in which area? - Member of a nursing profession (e.g. nurse, therapist, rescuer) | 18 | 64.3* |
|  |  | If yes, in which area? - Staff outside direct patient care (e.g. administrative staff, medical technicians, scientists) | 5 | 17.9* |
|  | Did you have sufficient time to think about the decision to consent? | Yes | 212 | 94.2 |
|  |  | No | 7 | 3.1 |
|  |  | Missing | 6 | 2.7 |
| Understanding | Did you understand the contents of the consent before you decided to consent? | Not at all | 1 | 0.4 |
|  |  | Mostly not | 2 | 0.9 |
|  |  | Yes, mostly | 81 | 36.0 |
|  |  | Yes, completely | 133 | 59.1 |
|  |  | Missing | 8 | 3.6 |
|  | Do you feel that the benefits of participating in this study have been explained? | Not at all | 1 | 0.4 |
|  |  | Mostly not | 9 | 4.0 |
|  |  | Yes, mostly | 81 | 36.0 |
|  |  | Yes, completely | 124 | 55.1 |
|  |  | Missing | 10 | 4.4 |
|  | Do you feel that the inconveniences and risks of participating in this study have been explained to you? | Not at all | 6 | 2.7 |
|  |  | Mostly not | 23 | 10.2 |
|  |  | Yes, mostly | 61 | 27.1 |
|  |  | Yes, completely | 124 | 55.1 |
|  |  | Missing | 11 | 4.9 |
|  | Did you feel that the study staff were available and willing to answer your questions or concerns about the study? | Not at all | 0 | 0.0 |
|  |  | Mostly not | 7 | 3.1 |
|  |  | Yes, mostly | 36 | 16.0 |
|  |  | Yes, completely | 173 | 76.9 |
|  |  | Missing | 9 | 4.0 |
|  | Were your questions and concerns addressed satisfactorily by the study staff? | Not at all | 0 | 0.0 |
|  |  | Mostly not | 2 | 0.9 |
|  |  | Yes, mostly | 40 | 17.8 |
|  |  | Yes, completely | 164 | 72.9 |
|  |  | Missing | 19 | 8.4 |
| Educational material | patient information - informed about it | Partly | 36 | 16.0 |
|  |  | Not at all | 7 | 3.1 |
|  |  | Not clear | 5 | 2.2 |
|  |  | Yes, completely | 164 | 72.9 |
|  |  | Missing | 13 | 5.8 |
|  | patient information - extent | Not detailed enough | 7 | 3.1 |
|  |  | Exactly right | 127 | 56.4 |
|  |  | Too detailed | 47 | 20.9 |
|  |  | I have not read it | 33 | 14.7 |
|  |  | Missing | 11 | 4.9 |
|  | patient information - understanding | Overall yes | 98 | 43.6 |
|  |  | Mostly yes | 79 | 35.1 |
|  |  | Mostly no | 0 | 0.0 |
|  |  | Overall no | 6 | 2.7 |
|  |  | I have not read it | 2 | 0.9 |
|  |  | Missing | 15 | 6.7 |
|  | educational video - informed about it | Partly | 12 | 5.3 |
|  |  | Not at all | 63 | 28.0 |
|  |  | Not clear | 7 | 3.1 |
|  |  | Yes, completely | 119 | 52.9 |
|  |  | Missing | 24 | 10.7 |
|  | educational video - extent | Not detailed enough | 7 | 3.1 |
|  |  | Exactly right | 108 | 48.0 |
|  |  | Too detailed | 13 | 5.8 |
|  |  | I have not seen it | 81 | 36.0 |
|  |  | Missing | 16 | 7.1 |
|  | educational video - understanding | Overall yes | 99 | 44.0 |
|  |  | Mostly yes | 28 | 12.4 |
|  |  | Mostly no | 1 | 0.4 |
|  |  | Overall no | 0 | 0.0 |
|  |  | I have not seen it | 81 | 36.0 |
|  |  | Missing | 16 | 7.1 |
| Personal reason not consented modules | I have not consented to any of the modules because | ... too little time to think about it. | 6 | 2.7 |
|  |  | ... I am generally not interested in donating data. | 6 | 2.7 |
|  |  | ... I have concerns about data protection. | 9 | 4.0 |
|  |  | ... I did not want to make this decision in the stressful medical situation of my emergency department stay. | 5 | 2.2 |
| Understanding | If you have consented to the data donation of your current treatment in the emergency department, which specifically does this concern? | Data on my treatment in the emergency department without data from the possibly following inpatient stay. | 16 | 7.1 |
|  |  | Data on my treatment in the emergency department and data from the possibly following inpatient stay. | 146 | 64.9 |
|  |  | I don't know. | 33 | 14.7 |
|  |  | Missing | 30 | 13.3 |
